# Genetic Diversity and *Wolbachia* Infection Patterns in a Globally Distributed Invasive Ant

**DOI:** 10.3389/fgene.2019.00838

**Published:** 2019-09-17

**Authors:** Shu-Ping Tseng, James K. Wetterer, Andrew V. Suarez, Chow-Yang Lee, Tsuyoshi Yoshimura, DeWayne Shoemaker, Chin-Cheng Scotty Yang

**Affiliations:** ^1^Research Institute for Sustainable Humanosphere, Kyoto University, Kyoto, Japan; ^2^Wilkes Honors College, Florida Atlantic University, Jupiter, FL, United States; ^3^Department of Evolution, Ecology and Behavior and Department of Entomology, University of Illinois Urbana-Champaign, IL, United States; ^4^Department of Entomology, University of California, Riverside, CA, United States; ^5^Entomology and Plant Pathology, University of Tennessee, Knoxville, TN, United States

**Keywords:** horizontal transfer, invasive species, phylogeography, selective sweep, *Wolbachia*

## Abstract

Understanding the phylogeographic history of an invasive species may facilitate reconstructing the history and routes of its invasion. The longhorn crazy ant, *Paratrechina longicornis*, is a ubiquitous agricultural and household pest throughout much of the tropics and subtropics, but little is known about the history of its spread. Here, we examine worldwide genetic variation in *P. longicornis* and its associated *Wolbachia* bacterial symbionts. Analyses of mtDNA sequences of 248 *P. longicornis* workers (one per colony) from 13 geographic regions reveal two highly diverged mtDNA clades that co-occur in most of the geographic regions. These two mtDNA clades are associated with different *Wolbachia* infection patterns, but are not congruent with patterns of nDNA (microsatellite) variation. Multilocus sequence typing reveals two distinct *Wolbachia* strains in *P. longicornis*, namely, *w*LonA and *w*LonF. The evolutionary histories of these two strains differ; *w*LonA appears to be primarily transmitted maternally, and patterns of mtDNA and nDNA variation and *w*LonA infection status are consistent with a relatively recent *Wolbachia*-induced selective sweep. In contrast, the observed patterns of mtDNA variation and *w*LonF infections suggest frequent horizontal transfer and losses of *w*LonF infections. The lack of nDNA structure among sampled geographic regions coupled with the finding that numerous mtDNA haplotypes are shared among regions implies that inadvertent long-distance movement through human commerce is common in *P. longicornis* and has shaped the genetic structure of this invasive ant worldwide.

## Introduction

Globalized human commerce has facilitated and intensified the spread of alien species, and the number of invasive species threatening native biodiversity, natural resources, and the economy continues to increase (Pimentel et al., 2000; Leppc et al., 2002; Occhipinti-Ambrogi and Savini, 2003; Meyerson and Mooney, 2007). Knowledge of the invasion histories, routes, and subsequent spread of invasive species provides important information for developing practical management strategies (Estoup and Guillemaud, 2010). Population genetic analyses on invasive species may provide insights into the introduction pathways and help us understand the mechanisms underlying the invasion success. Such analyses also may help define management objectives and assist policy makers in developing management, prevention, and restoration strategies (Abdelkrim et al., 2005; Le Roux and Wieczorek, 2009; Chadès et al., 2011; Cristescu, 2015).

The longhorn crazy ant, *Paratrechina longicornis* (Latreille, 1802) (Hymenoptera: Formicidae), is a widespread agricultural and household pest found throughout much of the tropics and subtropics in both the Old World and New World (Wetterer, 2008). A previous study demonstrated the occurrence of an extraordinary, double-clonal reproduction system in a population of *P. longicornis* from Thailand. In this population, queens are produced clonally from their mother, males are produced clonally from their fathers, and workers are produced sexually and characterized by an excess of heterozygosity (Pearcy et al., 2011). High heterozygosity of workers, close association with humans, and high adaptability in disturbed environments of this species may help explain to some extent how this ant spread rapidly around the world even prior to the 20^th^ century (Weber, 1939; Harris and Berry, 2005; Lester, 2005; Wetterer, 2008; Pearcy et al., 2011). While the precise native range of this ant has been a source of debate and remains uncertain, distribution records of *P. longicornis* and its closest relatives suggest either a Southeast Asian or African origin (Wetterer, 2008; LaPolla et al., 2010; LaPolla et al., 2013; LaPolla and Fisher, 2014). A comprehensive phylogeographic study of *P. longicornis* is needed to help identify more precisely where the species originated as well as its subsequent dispersal routes around the globe.

Researchers routinely analyze both mitochondrial DNA (mtDNA) and nuclear DNA (nDNA) data to address questions in molecular ecology and invasion biology. Typically, low genetic variation within a focal population is interpreted as resulting from one or more population bottlenecks after colonization. However, low mtDNA variation also can result from a recent “selective sweep” of a single, highly successful mtDNA variant, a process which may have no discernable effect on nDNA variation (Nei et al., 1975; Aquadro, 1997). This pattern also can stem from indirect selection associated with a selectively-favored, maternally-inherited symbiont (Hurst and Jiggins, 2005). Such symbionts are common in many insect populations and play a major role in shaping host mtDNA evolutionary history (Hurst and Jiggins, 2005; Moran et al., 2008; Charlat et al., 2009; Feldhaar, 2011; Richardson et al., 2012; Bennett and Moran, 2015; Schuler et al., 2016; Schuler et al., 2018). If a maternally-inherited symbiont confers a sufficient selective advantage to spread within and among host populations, the mtDNA variant originally associated with this symbiont may spread with it, and result in a skewed frequency distribution of mtDNA alleles during the process (Caspari and Watson, 1959; Kambhampati et al., 1992; Turelli et al., 1992; Narita et al., 2006; Atyame et al., 2011; Schuler et al., 2016). Several genera of such bacterial symbionts are found in insects, including *Wolbachia*, *Cardinium*, *Rickettsia*, *Spiroplasma*, and *Arsenophonus* (Duron et al., 2008; Engelstädter and Hurst, 2009). Among these, *Wolbachia* appears to be the most widespread maternally-transmitted symbiont in insects (Zug and Hammerstein, 2012; Weinert et al., 2015). *Wolbachia* variants typically spread within host species by increasing the relative fitness of infected females, either by conferring direct fitness benefits, such as increased fecundity (Vavre et al., 1999; Weeks et al., 2007; Zélé et al., 2018) or providing nutrients (Hosokawa et al., 2010; Nikoh et al., 2014), or by manipulating host reproduction *via* cytoplasmic incompatibility (CI), male-killing, feminization of genetic males, or thelytokous parthenogenesis (Werren et al., 2008; Saridaki and Bourtzis, 2010; Ma and Schwander, 2017). A relatively high proportion of ant species harbor *Wolbachia* infections (34%; Russell, 2012; Russell et al., 2012). Thus, possible symbiont effects on mtDNA variation in ants cannot be ignored. Incorporation of data from nuclear genes is essential to verify results obtained for mtDNA data because *Wolbachia* selective sweeps often, but not always, have little to no effects on nuclear variation (Rokas et al., 2001).

In this study we attempt to understand worldwide genetic variation and prevalence of *Wolbachia* in *P. longicornis*. We also assessed the geographic patterns of mtDNA variation in *P. longicornis*, to see if phylogeographic structure can help track the routes of dispersal of this invasive ant species. Our combined results allow us to test whether *Wolbachia* have exerted some selective pressure on mtDNA variation in *P. longicornis*. Also, patterns of mtDNA and nDNA variation were compared for incongruence, which would be predicted if mtDNA variation has been affected by co-evolving reproductive parasite. Lastly, because mtDNA genomes and endosymbionts are maternally co-inherited, analyses of mtDNA structure and variation can shed light on historical transmission patterns (e.g., potential source and spread) of endosymbionts in *P. longicornis*.

## Materials and Methods

### mtDNA Sequencing and Phylogenetic Analyses

We obtained *P. longicornis* workers from field collections and from other researchers (**Table S1**). A total of 248 ant colonies were sampled across the current geographic distribution of *P. longicornis*, including 22 colonies from Northeast Asia, 81 colonies from East Asia, 71 colonies from South Asia, 9 colonies from Indian Subcontinent, 17 colonies from Oceania, 9 colonies from Polynesia, 9 colonies from North America, 2 colonies from South America, 19 colonies from Caribbean, 2 colonies from Arabia, 2 colonies from Southeastern Europe, 4 colonies from West Africa, and 1 colony from South Africa. To generate statistically unbiased samples, only a single worker ant was used from each colony for subsequent genetic analyses. DNA was extracted from individual *P. longicornis* workers using the Gentra Puregene cell and tissue kit (Qiagen, USA) following the manufacturer’s instructions, and stored at −20°C. Portions of the cytochrome oxidase subunit I (COI, 1,203 bp), an intergenic spacer (106 to 127 bp), tRNA–Leu (70 to 77 bp), and the cytochrome oxidase subunit II (COII, 547 bp) genes were amplified *via* polymerase chain reaction (PCR). PCR was performed using the primer pair C1-J-1745M-F/PLCOII-R2 for partial COI and PLCOII-F1/C2-N-3661R for CO1-tRNA-COII region follow the PCR conditions described below (Degnan et al., 2004; **Table S2**). PCR mixtures contained 1–2 µL of template DNA, 0.2 µM of each primer, Takara EmeraldAmp Max PCR Master Mix (Takara, Japan) and water (20 µL reactions). PCR conditions included an initial denaturation step at 98°C (3 min) followed by 35 cycles of 94°C (30 s), 52°C (30 s), 72°C (2 min), and a final extension phase at 72°C (7 min). All PCR products were sequenced in both directions by Genomics BioSci and Tech Corp. (Taipei, Taiwan) using an ABI3730 sequencer. Sequence data were assembled using Sequencher 4.9 (GeneCodes).

Sequences were aligned using MUSCLE as implemented in MEGA 6 with default settings (Tamura et al., 2013). The intergenic spacer and tRNA–Leu region were excluded from phylogenetic analyses due to its ambiguous alignment. We performed phylogenetic analyses using two mtDNA datasets, one including all 248 *P. longicornis* workers and a second containing only a single representative sequence for each of the 43 mitochondrial haplotypes and plus two outgroup taxa, *P. zanjensis* and *P. ankarana* (45 OTUs). PartitionFinder 1.0.1 software (Lanfear et al., 2012) was used to determine the best fit substitution model and partitioning scheme based on Akaike information criterion (AIC) scores. PartitionFinder for our full dataset indicated the best scheme had four partitions: first position of COI and COII, second position of COI, third position of COI and COII, and second position of COII. The preferred evolutionary model for these four partitions were GTR + G, HKY + I, GTR + G, and F81, respectively (GTR = General Time Reversible; G = gamma distribution; HKY = Hasegawa-Kishino-Yano; I = proportion of invariable sites; F81 = Felsenstein 1981). For the singleton haplotype dataset (45 OTU), PartitionFinder suggested the best scheme had five partitions: 1) first position of COI, 2) second position of COI, 3) third position of COI and COII, 4) first position of COII, and 5) second position of COII. The preferred evolutionary model for these five partitions was GTR + G, HKY + I, GTR + G, HKY + G, and F81, respectively. These best schemes were used as priors for Bayesian phylogeny inference. A Bayesian phylogeny was reconstructed using MrBayes 3.2.1 (Ronquist et al., 2012). Two independent runs of 10^7^ generations with 4 MCMC (Markov Chain Monte Carlo) chains were conducted simultaneously, starting from random trees and resampling each tree every 1,000 generations. Posterior probabilities were obtained from the 50% majority-rule consensus of trees sampled after discarding the first 25% of sampled trees.

### Network Analysis and Neutrality Tests

A median joining mtDNA haplotype network was constructed using POPART (Leigh and Bryant, 2015; software available at: www.popart.otago.ac.nz) to infer relationships among haplotypes. Net genetic divergence between and within groups (p-distance) was estimated using MEGA 6 (Tamura et al., 2013). Population genetic parameters, including number of segregating sites *S* (Watterson, 1975), number of haplotypes *h*, haplotype diversity *Hd* (Nei, 1987), and nucleotide diversity π/bp (Nei, 1987), were estimated using DNASP v5.10 (Librado and Rozas, 2009). This software also was used to perform neutrality tests including Tajima’s *D* (Tajima, 1989), Fu and Li’s D* and F* tests (Fu and Li, 1993) and McDonald and Kreitman test (McDonald and Kreitman, 1991). A mtDNA sequence from *P. zanjensis* was used as the outgroup for neutrality tests. Negative values of Tajima’s *D*, Fu and Li’s D* and F* may reflect a recent population expansion, purifying selection, or genetic hitchhiking, whereas positive values generally reflect a population bottleneck, genetic structure and/or balancing selection. The McDonald and Kreitman test (M–K test) compares the ratio of fixed and polymorphic synonymous and nonsynonymous changes (McDonald and Kreitman, 1991). Additionally, the DHEW test (Zeng et al., 2007b) was performed to detect the signatures of positive selection and hitchhiking on host mtDNA as implemented in the DH program (Zeng et al., 2007a; Zeng et al., 2007b). The DHEW test we used was a compound test of Tajima’s *D* (Tajima, 1989), Fay and Wu’s *Hn* (Fay and Wu, 2000), and Ewens–Watterson test (Watterson, 1978), and is thought to be more powerful in detecting positive selection and more robust to historical demographic changes. *P*-values of the DHEW test were estimated using 100,000 replications of coalescent simulation using DH package (available online: http://zeng-lab.group.shef.ac.uk/wordpress/?page_id=28). Normalized Fay and Wu’s *Hn* (Fay and Wu, 2000; Zeng et al., 2006) was also calculated using the same package.

### Screening for *Wolbachia* Infection and MLST Sequencing

We screened the DNA samples for *Wolbachia* using three primer pairs that amplified part of the *Wolbachia* surface protein gene (*wsp*), 16S rRNA gene, and cell division protein (*ftsZ*) (**Table S3**). PCR primers are published elsewhere and listed in **Table S3**. PCRs were carried out for each of the three genes, and at least one *Wolbachia*-positive sample and deionized/distilled H_2_O were included as positive control and blank, respectively. Three workers per colony were used to determine the infection status for each colony. Our preliminary results indicated a high intra-colony infection rate of *Wolbachia* in both workers and queens (approximately 0.96–0.97, Tseng et al., unpublished data). All PCR amplicons that yielded a single band on agarose gels were sequenced by Genomics BioSci and Tech Corp. (Taipei, Taiwan) using an ABI3730 sequencer. Some workers appeared to be infected with multiple *Wolbachia* (see *Results* section for more details), and, in these cases, sequence data for individual *Wolbachia* was obtained by PCR using group- or strain-specific primers (**Table S4**).

Although *Wolbachia* surface protein (*wsp*) gene sequence data have been used for phylogenetic analyses in numerous studies, the phylogenetic relationships inferred using data based on a single gene may not be robust due to a high level of recombination among *Wolbachia* strains (Baldo and Werren, 2007). Therefore, we employed a multilocus sequence typing (MLST) approach developed by Baldo et al. (2006) in which a total of five MLST genes (*gatB*, *coxA*, *hcpA*, *ftsZ*, and *fbpA*) were sequenced following the methods of Baldo et al. (2006). *Wolbachia* strains were characterized by comparisons with other sequences in the *Wolbachia* MLST database (http://pubmlst.org/Wolbachia/) and NCBI Genbank database (https://www.ncbi.nlm.nih.gov/genbank/). ClonalFrame version 1.1 was used to construct a *Wolbachia* MLST genealogy (Didelot and Falush, 2007). ClonalFrame accounts for both substitutions and recombination events, providing more reliable clonal relationships based on multilocus data (Didelot and Falush, 2007). Two independent runs were performed, each with 1,000,000 MCMC burn-in iterations and 1,000,000 as sampling period and a sampling frequency of 1,000. A 50% majority rule consensus tree was built from combined data from the two independent runs.

### Reconstruction of Ancestral States of *Wolbachia* Infection Status

The program BayesTraits was utilized to reconstruct the ancestral states of *Wolbachia* infections in *P. longicornis* mtDNA lineages (Pagel et al., 2004; Mark and Andrew, 2006). This software considers uncertainties in estimating a tree and its branch lengths when inferring ancestral states, and thus may provide different inference results from the parsimonious expectation. Two *Wolbachia* strains, *w*LonA and *w*LonF, were found in some of our *P. longicornis* samples (see *Results* section for details). We tested for a correlation between the occurrence/absence of *w*LonA and the occurrence/absence of *w*LonF by performing BayesTrait analyses using both dependent (i.e., the infection history of *w*LonA was correlated with *w*LonF) and independent models. The difference between the two models was assessed by Bayes Factor (BF) based on the final harmonic mean of the likelihoods model. A log BF value greater than two was interpreted as supporting the dependent model (i.e., correlated patterns of infections). Prior to the MCMC runs, maximum likelihood analyses were performed using the consensus tree obtained from MrBayes, and the derived results were used to set the priors for MCMC analyses. Considering the results of the likelihood analysis, all MCMC priors were set as uniform distribution for all rates, with different ranges used for each parameter. A total of 7,500 trees were generated by MrBayes (full dataset with 248 OTUs, discarded first 25% trees as burn-in) and used in the MCMC inferences to account for phylogenetic uncertainty. These input trees did not include an outgroup species because we focused only on infection histories of the two *Wolbachia* strains in *P. longicornis*. Terminal taxa were coded for presence (1) or absence (0) of *Wolbachia* infection. The rate deviation parameter was tuned automatically to achieve an average acceptance rate between 20% and 40% and ancestral states were reconstructed using the command “addnode”. The MCMC chains were run for 10^9^ iterations, sampled every 10^5^ iterations with a burn-in of 10^8^ iterations.

We tested for associations between mitochondrial lineages and *Wolbachia* infection status by using the BaTS program (Bayesian tip-association significance testing) to compute the parsimony score statistic of clustering strength (PS), the association index statistic (AI), and the exclusive single-state clade size statistic (MC) (Parker et al., 2008). PS represents the most parsimonious number of character changes in the phylogeny. AI is an estimate of the frequency of the most common branch tip trait subtended by internal nodes. MC measures the size of the maximum monophyletic clade in which all tips share the same trait. Thus, a significantly lower value of PS, lower AI and higher MC would indicate a strong phylogeny–trait association. The association between mitochondrial lineage and *Wolbachia* infection is predicted to be strong if *Wolbachia* infections are transmitted vertically only, while frequent horizontal transfers of *Wolbachia* infections would erode this association. In BaTS analyses, 7,500 trees were generated by MrBayes as input and tested each parameter by generating a null distribution from 1,000 replicates.

### nDNA Analyses

We genotyped a subset of *P. longicornis* workers from three well-sampled regions (41 colonies from East Asia, 21 colonies from Northeast Asia, and 71 colonies from South Asia) at 20 microsatellite loci (Tseng et al., 2019) (**Table S5**) to test for congruence (or incongruence) of mtDNA and nuclear DNA variation patterns. DNA of the same worker (one worker per colony) was used for both mtDNA and microsatellite analyses. Microsatellite loci were amplified by using a multiplex PCR method following procedures described by Blacket et al. (2012). The purified PCR products were analyzed on an ABI-3730 Genetic Analyzer (Applied Biosystems) by Genomics BioSci and Tech Co., Ltd (Taipei, Taiwan). GeneMarker (version 2.4.0, Softgenetics LLC) was employed to visualize and score alleles. Genetic variation at each microsatellite locus was characterized in terms of number of alleles (*Na*), effective number of alleles (*Ne*), observed (*Ho*) and expected (*He*) heterozygosity, Shannon’s information index (*I*), fixation index (*F*), and Hedrick’s standardized *G_st_* for small number of populations (*G’’*_ST_), using the program GENALEX 6.502 (Peakall and Smouse, 2006).

Genetic structure was assessed using the Bayesian model-based clustering software STRUCTURE 2.3.4 (Pritchard et al., 2000; Hubisz et al., 2009). Five independent STRUCTURE runs were executed for each of *K* = 1–10 (*K*, the number of assumed genetic clusters) under the admixture model and allele frequencies correlated with 1,000,000 MCMC iterations and an initial burn-in of 100,000 generations. The optimal number of genetic clusters within the data was estimated by Evanno et al. (2005) in STRUCTURE HARVESTER v. 0.9.94 (Earl and vonHoldt, 2012) (available online: http://taylor0.biology.ucla.edu/structureHarvester/). STRUCTURE results were visualized using CLUMPAK server (Jakobsson and Rosenberg, 2007) (available online: http://clumpak.tau.ac.il/). Genetic relationships among populations were examined by applying a discriminant analysis of principal components (DAPC) (Jombart et al., 2010) available in the R (R Core Team, 2014) package adegenet (Jombart, 2008) on all microsatellite data. Population labels were input as the prior cluster information in DAPC. The first 20 principal components (PCs) accounted for 80% of the total microsatellite genetic variation and were retained from the analysis.

## Results

### mtDNA Analyses

**Figure 1:**
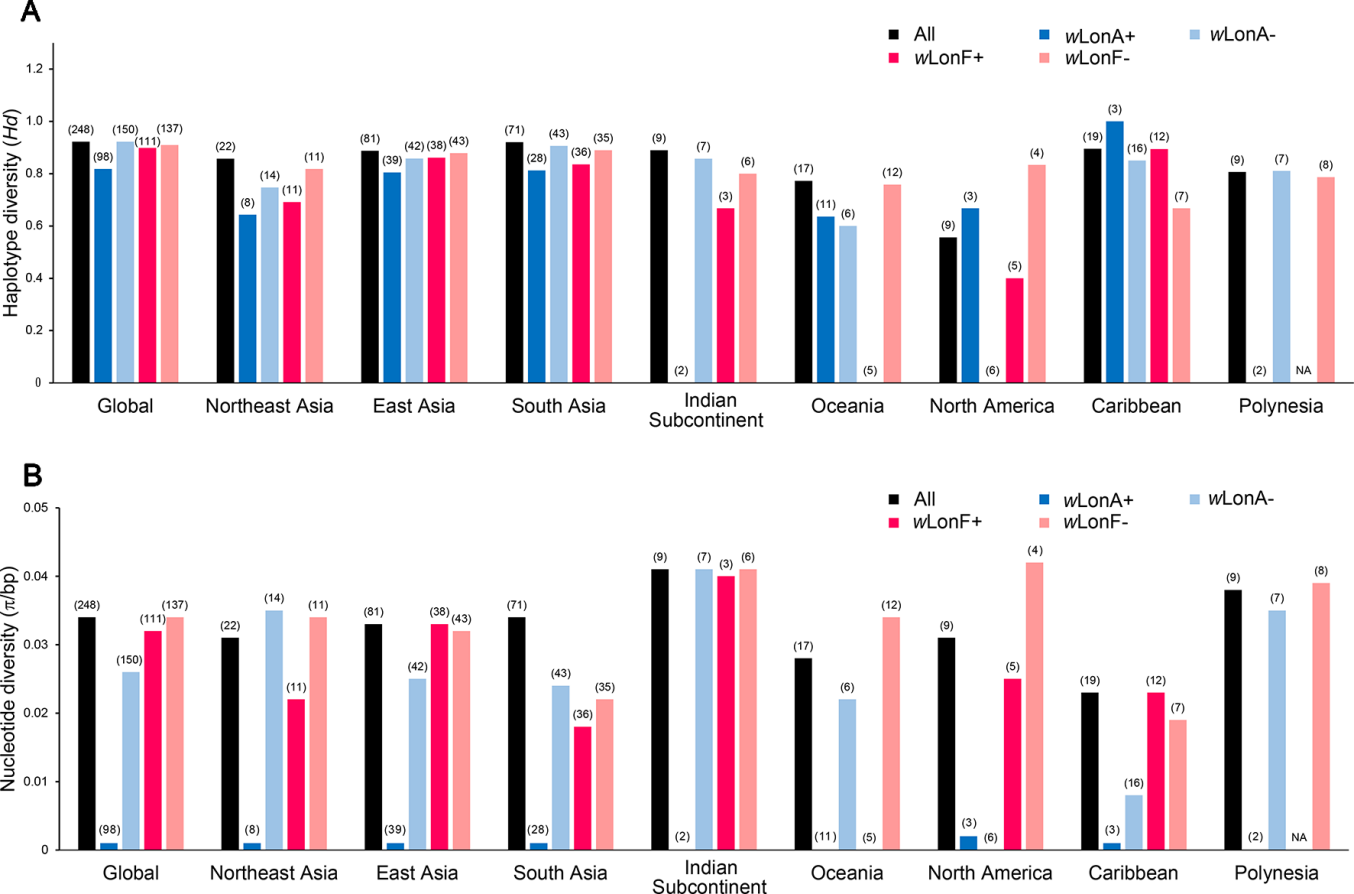
Regional mitochondrial genetic diversity of *Paratrechina longicornis* as expressed by **(A)** haplotype diversity and **(B)** nucleotide diversity with respect to their *Wolbachia* infection status. *w*LonA+, *w*LonA−, *w*LonF+, and *w*LonF− denote *w*LonA-infected, *w*LonA-uninfected, *w*LonF-infected, and *w*LonF-uninfected ants in a given region, respectively. Sample size of each region is indicated in parentheses.

**Figure 2:**
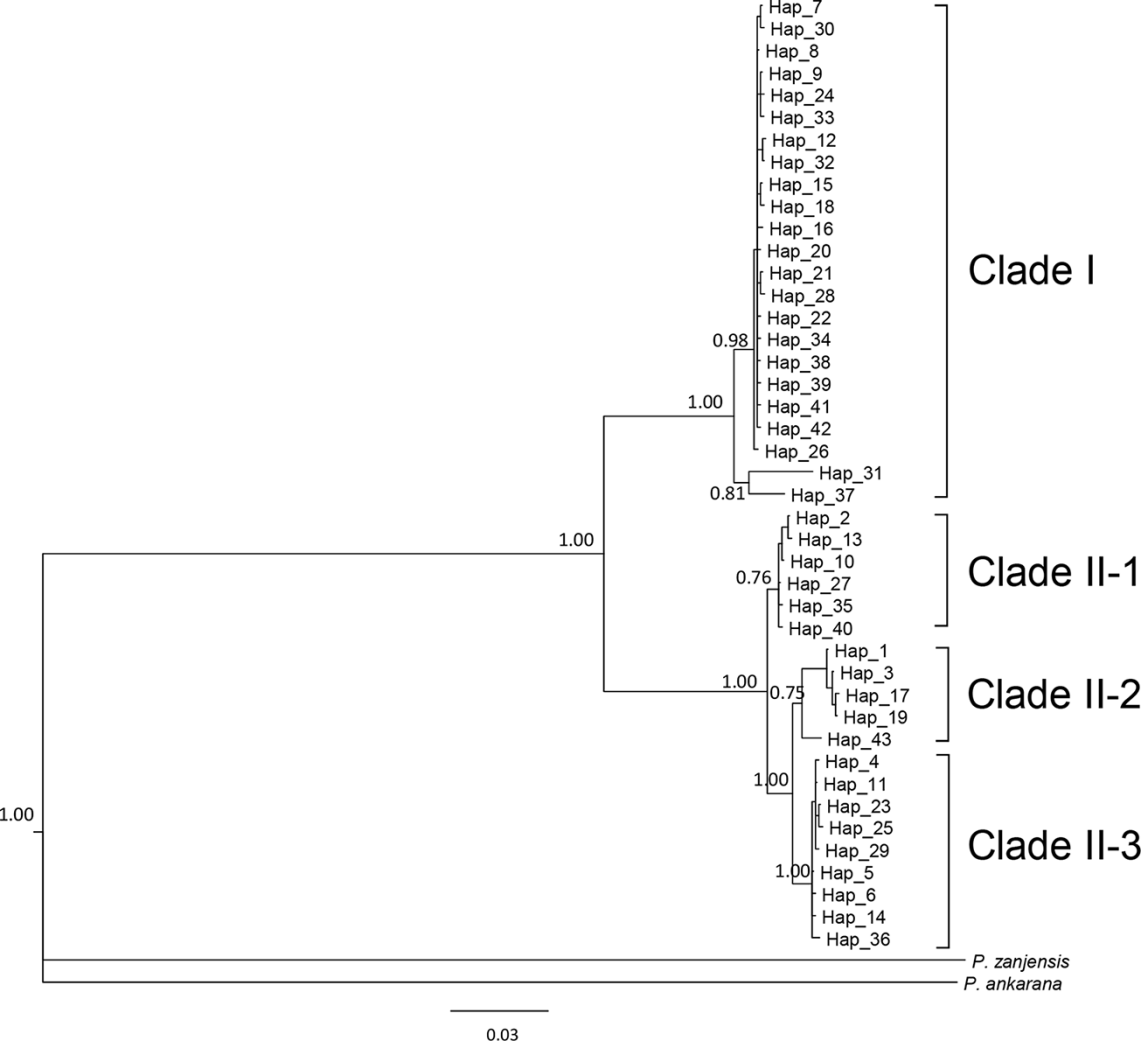
The 50% majority rule consensus tree for all sampled *Paratrechina longicornis*, inferred by Bayesian analysis. Numbers above branches indicate Bayesian posterior probability calculated by MrBayes. Refer to **Table S1** for respective geographic information of each haplotype.

**Figure 3:**
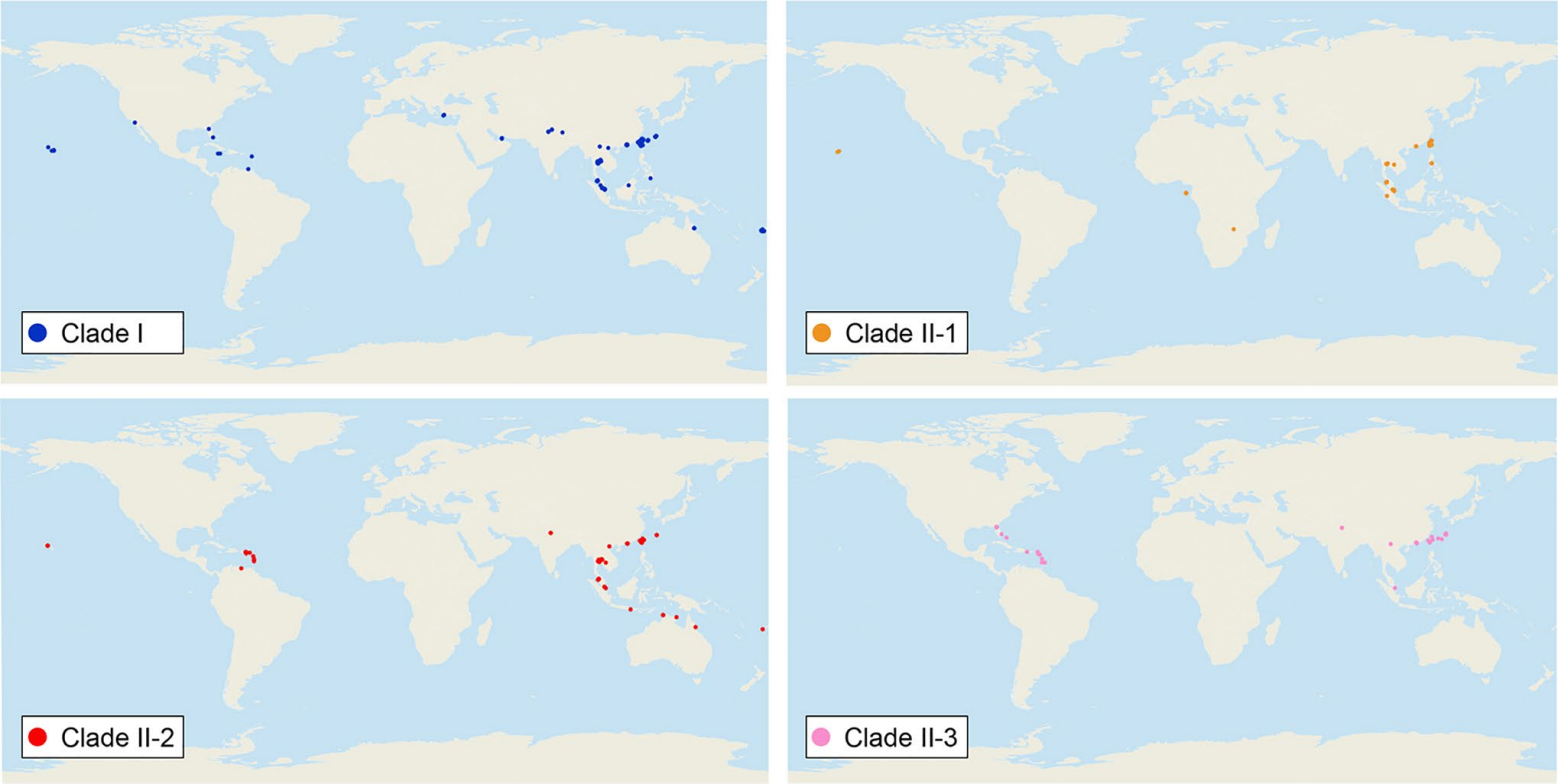
Distribution of all mitochondrial haplogroups in the study regions. Haplogroups are denoted by colors: Clade I (blue), Clade II-1 (orange), Clade II-2 (red), and Clade II-3 (pink).

**Figure 4:**
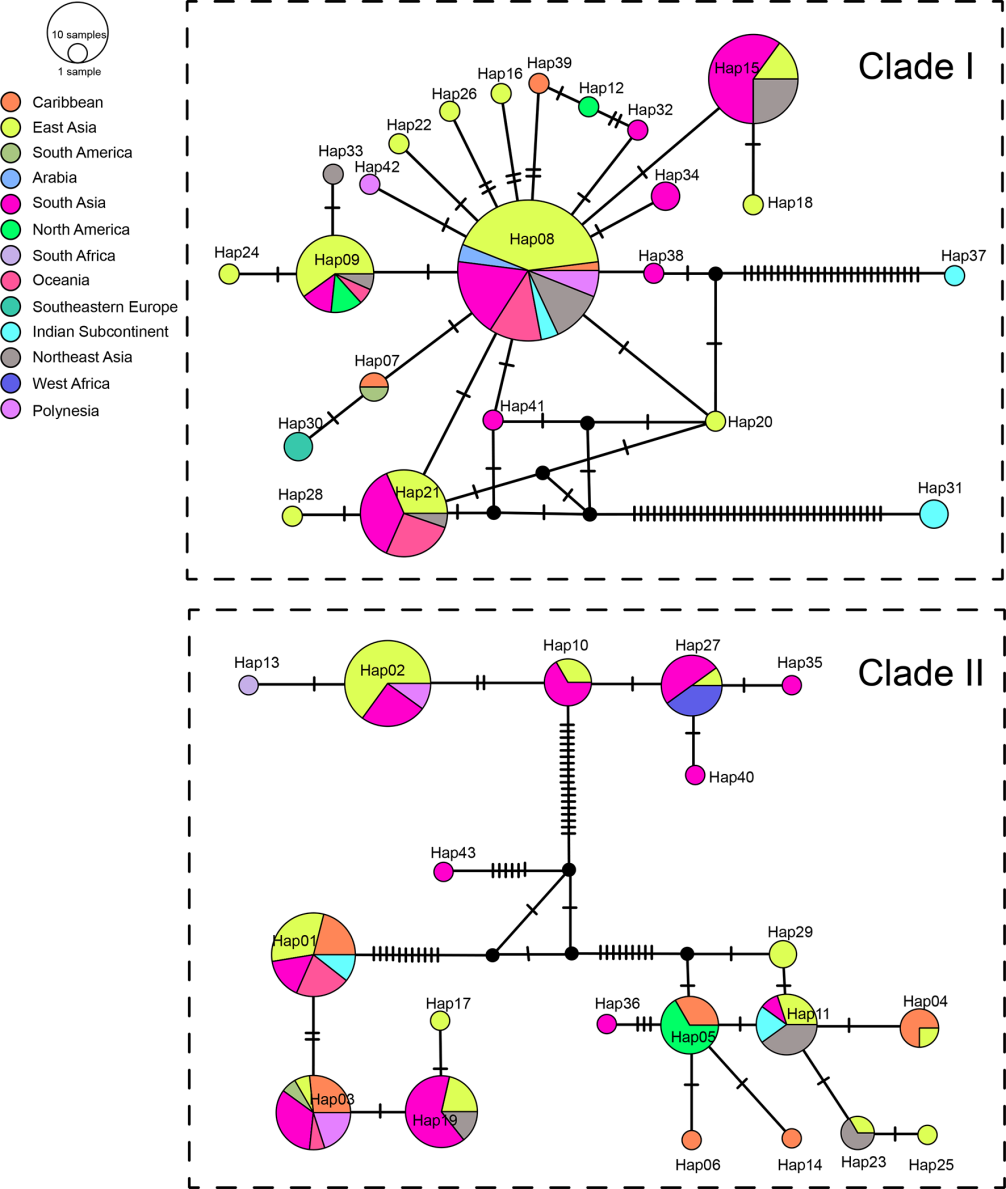
Haplotype networks of the mitochondrial genes. Circle sizes are proportional to the number of sequences per haplotype. Colors correspond to geographic regions.

We sequenced mtDNA of 248 *P. longicornis* workers (one per colony) from 13 geographic regions. A total of 43 different mtDNA haplotypes were found with 172 polymorphic sites present over the entire 1,750bp COI-COII region (**Table S6**; collection site information in **Table S1**). Nucleotide diversity was highest in samples from the Indian Subcontinent (0.041) (**Figure 1**; **Table S6**) genetic diversity values from Arabia, Southeastern Europe, West Africa, and South America are likely biased due to low sample size). Nevertheless, the populations across Old World regions exhibit similar levels of genetic diversity. Bayesian phylogenetic analyses indicated the presence of two mtDNA clades (Clade I and II), one of which (Clade II) was divided into three subclades (Clade II-1, -2 and -3) (**Figure 2**). The average genetic distance between Clades I and II was 0.057, suggesting deep divergence between the two clades. The average pairwise genetic distance among haplotypes was higher within Clade II (0.010) compared with Clade I (0.002). Average genetic distances among workers within the three subclades each had a mean value of 0.001. MtDNA variation was not strongly correlated with geographic location (**Figure 3**). Workers belonging to Clades I and II were found at 11 of the 13 sampled geographic regions (all except South and West Africa; **Figure 3**). Workers with haplotypes belonging to subclade II-1 were found in the Old World, but not in the New World (**Figure 3**). The median-joining network constructed for all 43 unique mtDNA haplotypes further revealed no clear spatial clustering of haplotypes from Clade I (**Figure 4**). In particular, haplotype Hap08 (Clade I) was common across the sampled ranges and was connected to several tip haplotypes with low frequency, implying that this haplotype may represent a putative ancestral haplotype within Clade I from which the latter are derived. Similar to Clade I, the haplotype network revealed negligible spatial clustering in Clade II (**Figure 4**), with approximately half of all haplotypes in this clade present in more than one geographic region.

### *Wolbachia* Infections in *P. longicornis*

Both sequence data and phylogenetic analyses of concatenated MLST data suggest that two *Wolbachia* strains, *w*LonA and *w*LonF, occur in *P. longicornis*, with the former belonging to supergroup A and the latter to supergroup F (**Figure S1**). Forty-two of the 248 *P. longicornis* workers were infected with *w*LonA only (17%), 55 workers were infected with *w*LonF only (22%), 56 workers were co-infected with *w*LonA and *w*LonF (23%), and 95 workers were uninfected (38%) (**Table 1**). *Wolbachia* infection status was strongly associated with mtDNA variation. Specifically, the majority of ants belonging to Clade I were either infected with *w*LonA only (33%) or co-infected with *w*LonA and *w*LonF (44%), whereas none of workers belonging to Clade II was infected with *w*LonA (**Table 1**). We did not observe a significant association between *Wolbachia* infection status and host geographic range (**Figure S2**).

**Table 1:** Prevalence of *Wolbachiaw*LonA and *w*LonF infections in *Paratrechina longicornis*.

No. nests (percentage) ^†^	*w*LonA	*w*LonAF	*w*LonF	Uninfected
Clade I	42 (33%)	56 (44%)	8 (6%)	21 (17%)
Clade II	0 (0%)	0 (0%)	47 (39%)	74 (61%)
Total	42 (17%)	56 (23%)	55 (22%)	95 (38%)

^†^Three workers from each nest were used to screen for Wolbachia infections.

The MLST allelic profile for *w*LonA was identical to a sequence type in the *Wolbachia* MLST database, whereas *w*LonF represented a new sequence type that has not been reported in the database. *w*LonA allelic proﬁles for *gatB*, *coxA*, *hcpA*, *ftsZ*, *fbpA* and *wsp* were 7, 6, 7, 3, 8, and 18, respectively (**Table S7**). *w*LonA belongs to sequence type 19 (ST-19), and is similar to *Wolbachia* variants detected in a moth (*Ephestia kuehniella*), several ants (*Technomyrmex albipes*, *Leptomyrmex* sp., *Pheidole plagiara*, *Ph. sauberi*, and *Leptogenys* sp.) and two butterflies (*Ornipholidotos peucetia* and *Aricia artaxerxes*) (**Table S7**). *w*LonA shared an identical sequence type with *Wolbachia* Ekue_A (ID 13) detected from *Ephestia kuehniella*, a transinfected A group *Wolbachia* from *Cadra cautella* (*w*CauA) to *E. kuehniella*. *Wolbachiaw*CauA has been reported to cause cytoplasmic incompatibility (CI) in *C. cautella*, and male killing in *E. kuehniella* (Sasaki et al., 2002; Sasaki et al., 2005). *w*LonF allelic proﬁles for *gatB*, *coxA*, *hcpA*, *ftsZ*, *fbpA* and *wsp* were 168, 147, 262, 132, 226, and 708, respectively (**Table S7**), and were unique to consider *w*LonF a new sequence type (denoted as ST-471). Similar sequence types included ST-239, ST-242, and ST-243, all of which were detected from two dragonflies (*Brachythemis contaminata* and *Orthetrum sabina*). The most similar *wsp* sequence to *w*LonF in GenBank was from a *Wolbachia* variant infecting bat flies *Cyclopodia dubia* (KT751165; 99% similarity) (Wilkinson et al., 2016).

### *Wolbachia* Infection History in *P. longicornis*

**Figure 5:**
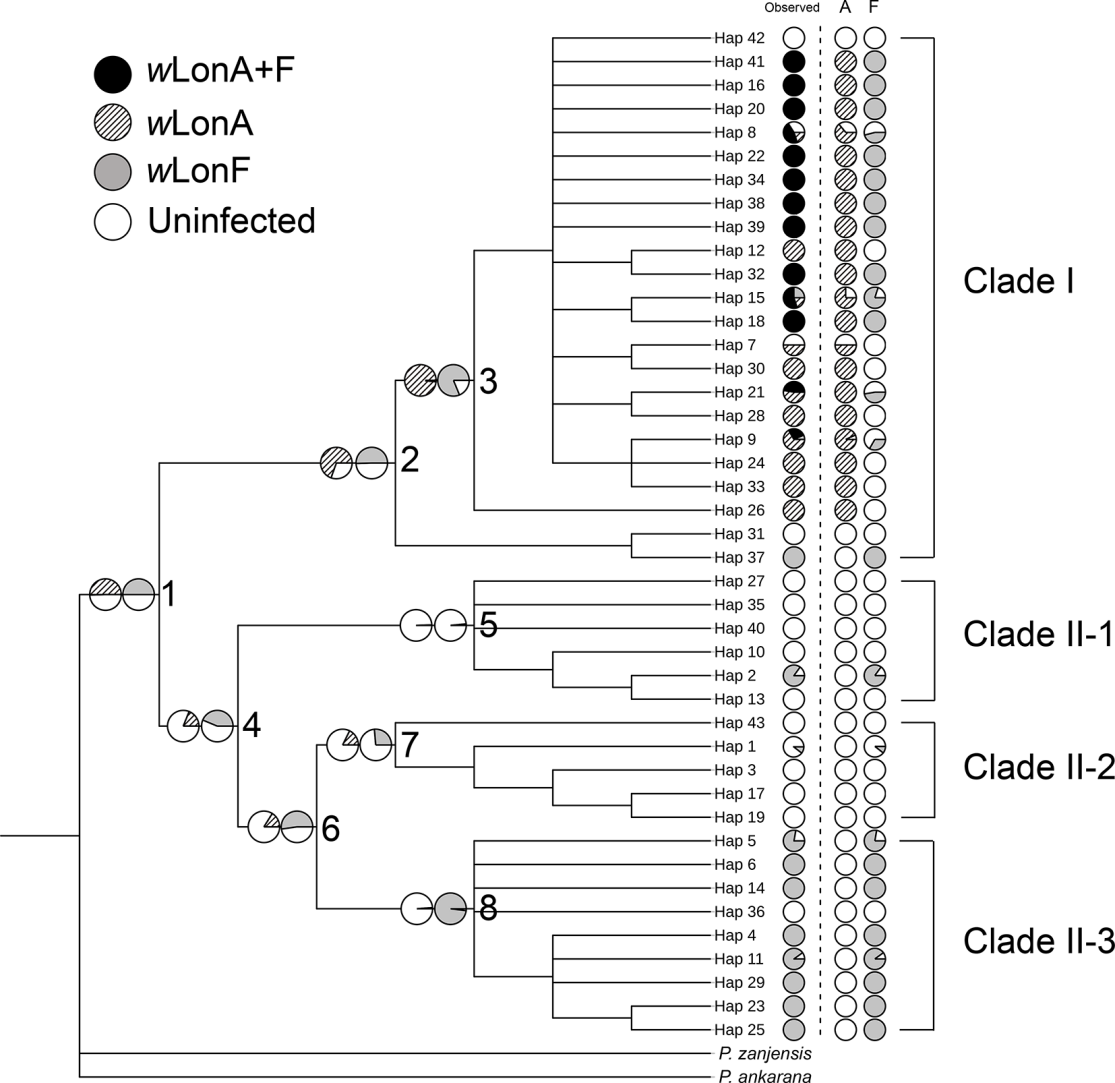
Simulated ancestral states of *Wolbachia* infection in *Paratrechina longicornis* inferred by BayesTraits. For each haplotype, pie charts following the haplotype name indicate observed *Wolbachia* infection status combined, *w*LonA only and *w*LonF only (*w*LonA+F co-infection: black; *w*LonA infected: upward diagonal; *w*LonF infected: grey; lack of infection: white). Pie charts on branches indicate simulated probabilities of *Wolbachia* infected status (left: *w*LonA; right: *w*LonF) for each numbered node.

The BayesTraits analyses indicated the dependent model was not significantly better than the independent model (log Bayes factors = 0.707), suggesting no correlation between *w*LonA infection status and *w*LonF infection status. Therefore, infection history of *w*LonA and *w*LonF was inferred separately (**Figure 5**). BayesTraits analyses suggested a single ancestral *w*LonA infection (on the common ancestor of node 2 or node 3, **Figure 5**) occurred in *P. longicornis* that subsequently has been characterized by vertical *Wolbachia* transmission in the populations of Clade I with only occasional losses of infections (**Figure 5**). On the other hand, the history *w*LonF infections in *P. longicornis* appears to be characterized by frequent gains of *w*LonF through horizontal transmission as well as frequent losses of *w*LonF over time. Although the association between *w*LonF infection status and host mtDNA phylogeny was weaker than that of *w*LonA (both AI and PS values of *w*LonF were higher than those of *w*LonA), the BaTS results indicated that both *w*LonA and *w*LonF are significantly associated with the host mtDNA phylogeny (**Table 2**). These results suggest that *w*LonF infection within *P. longicornis* has been shaped by both horizontal and vertical transmission. For example, individuals bearing haplotype 2 (Hap 2) likely obtained *w*LonF *via* horizontal transmissions whereas the high prevalence of *w*LonF in Clade II-3 is consistent with vertical transmission of *w*LonF over time (**Figure 5**).

**Table 2:** Significance of correlations between *Paratrechina longicornis* mtDNA phylogeny and *Wolbachia* infection status as identified by BaTS. Association index statistic (AI) and parsimony score (PS) statistic of clustering strength, and exclusive single-state clade size (MC) statistic.

Statistics	Observed mean (95% CI)	Null mean (95% CI)	*P*-value
*w*LonA			
AI	3.10 (2.03, 4.19)	10.46 (9.82, 11.06)	<0.0001
PS	24.29 (22.00, 27.00)	77.69 (74.31, 80.41)	<0.0001
MC (uninfected)	121.00 (121.00, 121.00)	5.63 (5.03, 6.50)	0.001
MC (infected)	19.89 (14.00, 24.00)	3.46 (3.17, 4.26)	0.001
*w*LonF			
AI	6.37 (4.87, 7.89)	10.82 (10.17, 11.47)	<0.0001
PS	49.67 (45.00, 54.00)	81.54 (77.87, 84.61)	<0.0001
MC (uninfected)	33.40 (30.00, 42.00)	4.97 (4.45, 5.71)	0.001
MC (infected)	10.56 (5.00, 18.00)	3.90 (3.55, 4.57)	0.001

### Patterns of mtDNA Variation Within *Wolbachia*-Infected and -Uninfected Ants

*w*LonA and *w*LonF may have undergone independent invasion histories in *P*. *longicornis* (**Figure 5**), therefore *w*LonA (or F)-positive individuals (including individuals with co-infection) are grouped together when mtDNA variation was analyzed to show the effect of a single strain on the mtDNA evolution. Analyses of mtDNA variation revealed that nucleotide diversity and numbers of segregating sites are much lower in *w*LonA-infected workers than those in *w*LonA-uninfected workers within all sampled regions, except North America (**Figure 1**; **Table S6**). Estimates of nucleotide diversity were more than 8-fold lower for *w*LonA-infected workers than in *w*LonA-uninfected workers despite limited differences in mtDNA variation between *w*LonF-infected workers and *w*LonF-uninfected workers.

**Figure 6:**
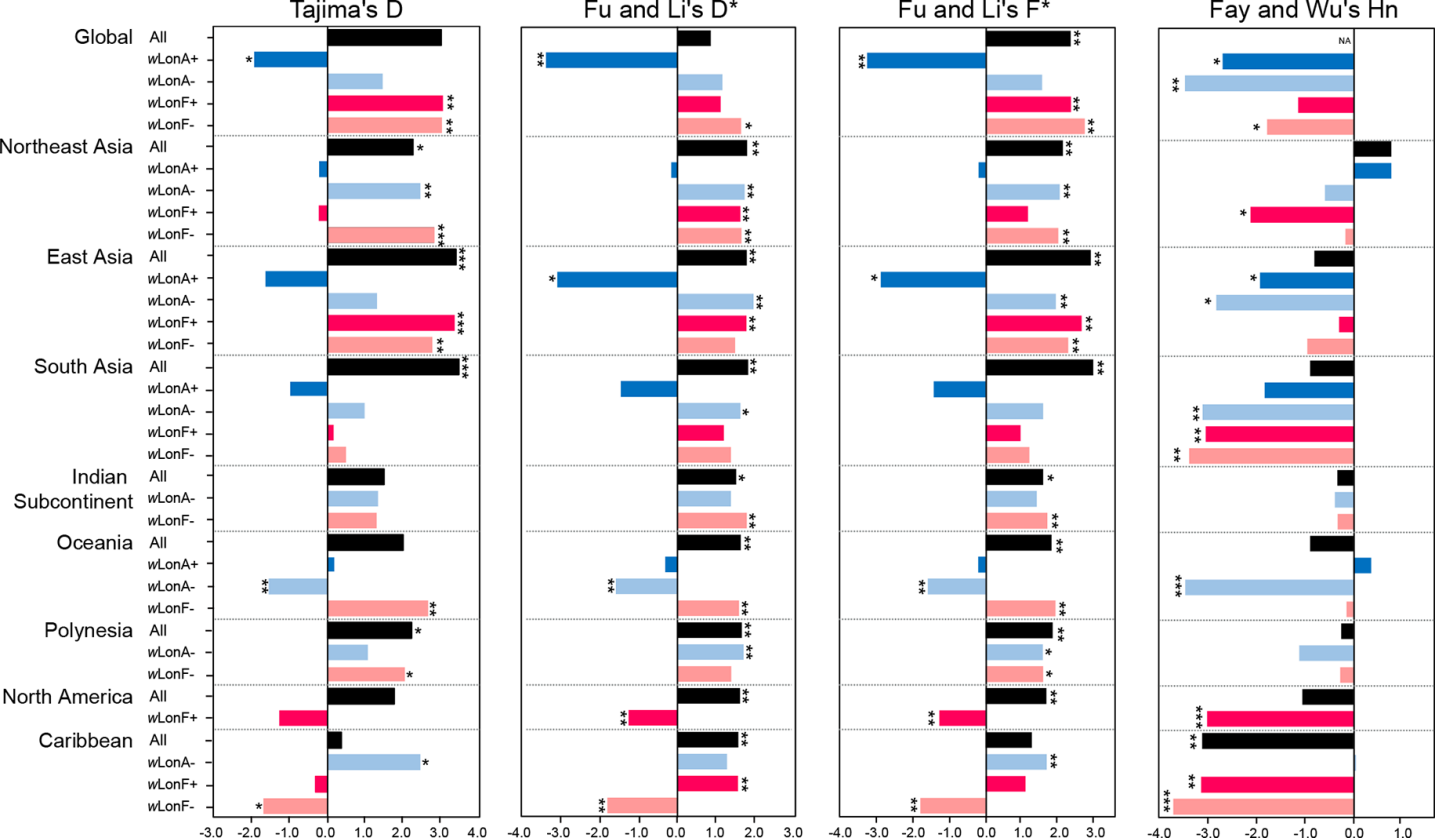
Tests for departure from neutrality for mtDNA sequence variation in *Paratrechina longicornis*. *w*LonA+, *w*LonA−, *w*LonF+, and *w*LonF− denote *w*LonA-infected, *w*LonA-uninfected, *w*LonF-infected, and *w*LonF-uninfected ants in a given region, respectively. *P < 0.05; **P < 0.01; ***P < 0.0001; statistics significantly deviated from expectations under neutrality.

Statistical tests of departures from neutral expectations are presented in **Table S8** and **Figure 6**. Estimates of Tajima’s *D*, Fu and Li’s D*, Fu and Li’s F*, and Fay and Wu’s *Hn* were negative and statistically significant when all *w*LonA-infected workers were combined (Global group; **Table S8**; **Figure 6**). The result of the DHEW test on the combined *w*LonA-infected workers supported the hypothesis of selection influencing mtDNA variation (**Table S8**). The neutrality index (NI) of the McDonald–Kreitman (M–K) test was greater than one and deviated from neutral expectations (**Table S8**). In contrast, Tajima’s *D*, Fu and Li’s D*, Fu and Li’s F* tests performed on combined samples in other three groups (*w*LonA-uninfected, *w*LonF-infected and *w*LonF-uninfected workers) were all positive, but only five of these estimates were significant (**Table S8**; **Figure 6**). The estimates of Fay and Wu’s *Hn* in *w*LonA-uninfected and *w*LonF-uninfected workers were negative and significantly less than zero. However, the results of DHEW tests failed to support the presence of positive selection of these two groups. The results of M–K tests indicated that the NI of these three groups were not significantly different from one.

Similar trends were found for estimates of Tajima’s *D*, Fu and Li’s D*, and Fu and Li’s F*statistic for all groups harboring *w*LonA (i.e., generally less than zero). However, the normalized *Hn* statistics of Fay and Wu test and results of DHEW varied among regions (**Table S8**). We obtained a negative yet significant estimate of *Hn* only for *w*LonA-infected workers from East Asia, and the results of DHEW tests for *w*LonA-infected groups from East Asia and South Asia regions were significant. The NI estimates from M–K test were larger than one for all groups harboring *w*LonA, and were significant in groups from Northeast and East Asia.

Tajima’s *D*, Fu and Li’s D*, Fu and Li’s F* generally were positive for *w*LonA-uninfected, *w*LonF-infected and *w*LonF-uninfected groups for each region with few exceptions. The significant negative estimates of *D*, D*, F*, *Hn* and significant results of DHEW test were observed in three groups, *w*LonA-uninfected workers from Oceania, *w*LonF-infected workers from North America, and *w*LonF-uninfected workers from Caribbean.

### Nuclear DNA Variation and Population Genetic Structure

**Figure 7:**
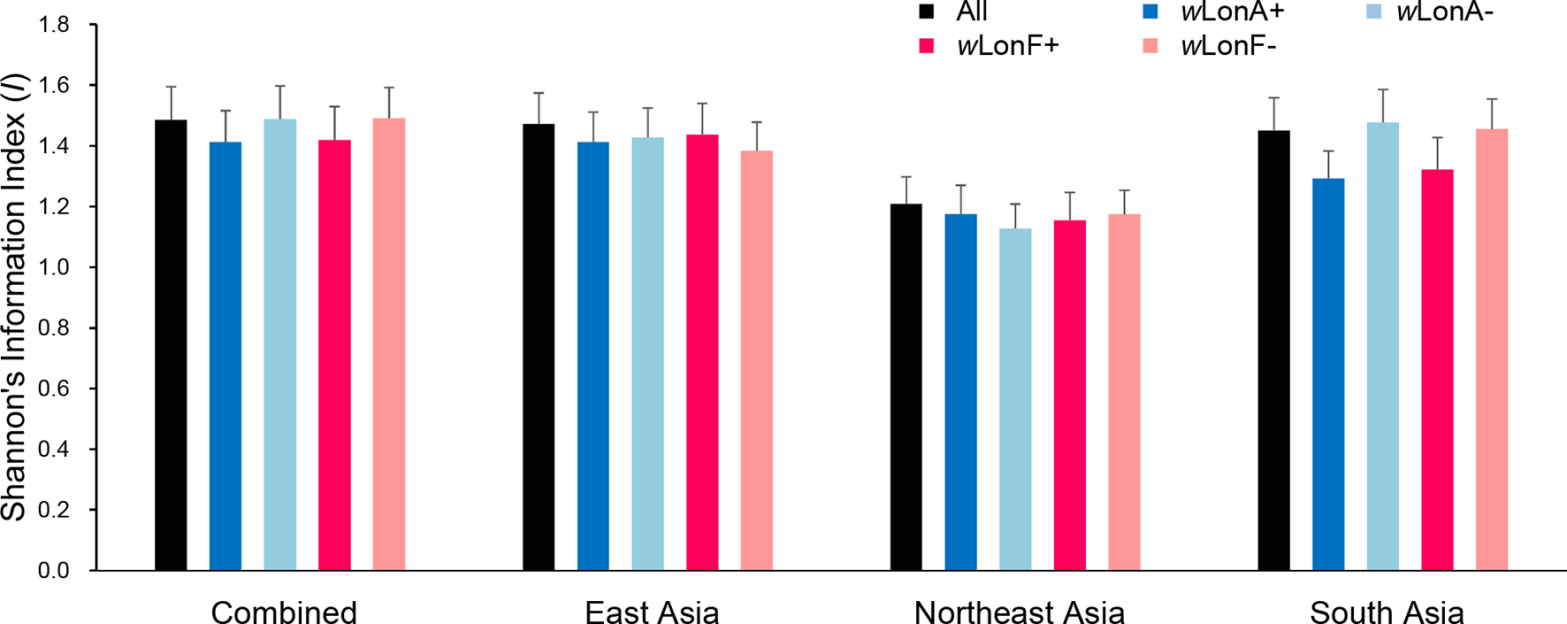
Genetic diversity, as expressed by Shannon’s information index, of *Paratrechina longicornis* in selected regions based on 20 microsatellite markers. *w*LonA+, *w*LonA−, *w*LonF+, and *w*LonF− denote *w*LonA-infected, *w*LonA-uninfected, *w*LonF-infected, and *w*LonF-uninfected ants in a given region, respectively. Error bars indicate standard errors.

We compared the extent of mtDNA and nuclear (microsatellite) differentiation in the three selected Asia regions. A total of 191 alleles were observed across all loci for the 134 sampled workers. The average number of alleles in each sampled region ranged from 4.85 to 8.10 (**Table S9**). Shannon’s information index was used to assess gene diversity (**Figure 7**; **Table S9**), and, when incorporating infection status into analysis, genetic diversities among *Wolbachia*-infected workers are similar to those for uninfected workers across the three selected regions.

Bayesian cluster analysis performed using STRUCTURE revealed six distinct genetic clusters for the entire data set (*K* = 3 based on ∆*K* statistic; **Table S10**). Most workers were admixed (i.e., had membership in more than one cluster; **Figure S3**), and genetic differentiation among geographic regions or mtDNA clades was not observed. DAPC analysis indicated the lack of differentiation among groups in each of the three selected regions as well as between the two mtDNA clades (**Figure S4**). The estimate of *G’’*_ST_ between the two mtDNA clades was 0.049 (*P* = 0.001), suggesting a low level of nuclear differentiation between workers from the two mtDNA clades.

## Discussion

Our results showed that global patterns of mtDNA and nDNA variation among populations of *P. longicornis* were discordant, characterized by two highly divergent mtDNA clades with no parallel pattern of nuclear genetic divergence (based on microsatellite loci) between workers from the two mtDNA clades. Several evolutionary scenarios possibly explaining such mitochondrial-nuclear discordance include sex-biased dispersal, local adaptation, historical demography, incomplete lineage sorting, and endosymbiont-driven hitchhiking effects (reviewed in Toews and Brelsford, 2012). Male-biased dispersal (López-Uribe et al., 2014) and local adaptation of mtDNA haplotypes (Cheviron and Brumfield, 2009; Ribeiro et al., 2011; Spottiswoode et al., 2011) are unlikely for at least two reasons: 1) the geographical distributions of ants from the two mtDNA clades overlap considerably and coexist in virtually all geographic regions we sampled and 2) all ant samples were collected from human-modified habitats (e.g., roadsides, parks or near buildings), suggesting negligible habitat preference between ants from the two clades. The strong association between *Wolbachia* infection status and host mtDNA lineage, as well as reduced mtDNA diversity associated with *w*LonA in *P. longicornis*, are consistent with *Wolbachia* influencing patterns of host mtDNA structure and variation. Levels of nuclear variation were nearly identical for ants from the *w*LonA-infected workers (mtDNA Clade I) and uninfected groups (mtDNA Clade II), which is consistent with the prediction that *Wolbachia* endosymbionts have minimal or no effects on nuclear genetic variation and divergence (assuming host reproduces sexually) due to biparental inheritance (Rokas et al., 2001).

Results from additional analyses also were largely consistent with the predicted effects of *Wolbachia* on mtDNA variation. Tajima’s D, Fu and Li’s D*, and Fu and Li’s F* tests for departures from neutral evolution were negative for all groups harboring *w*LonA and for global datasets. Moreover, the results of Fay and Wu’s *Hn* and DHEW were also consistent with expected patterns for a relatively recent *Wolbachia*-driven selective sweep occurring in some, but not all, geographic regions, such as East and South Asia, despite the fact that mtDNA variation is low in almost all populations harboring *w*LonA in every geographic region. One potential explanation for these inconsistencies is that selective sweeps of *Wolbachia* in some of these populations may have occurred far enough in the distant past such that any signature of selection on the mtDNA may have been eroded. The results of M–K tests also imply mtDNA substitution patterns may have been influenced by *w*LonA, but surprisingly that a signature of purifying selection is registered. The NI values of M–K test for groups harboring *w*LonA ranged between 5,074 to 12,888, and were significantly larger than values for groups from Northeast and East Asia. One possible explanation is that hitchhiking events associated with *w*LonA resulted in accumulation of slightly deleterious mutations (Shoemaker et al., 2000; Fay and Wu, 2000) followed by negative selection as *Wolbachia*-driven haplotype replacements cease (Bazykin and Kondrashov, 2011).

The virtual absence of genetic structure across a large geographic area in *P. longicornis* and the co-occurrence of divergent mtDNA haplotypes in almost every geographic region suggest that human-mediated, long-distance movement of this species is common. The time of divergence between any random pair of *w*LonA-uninfected groups (i.e., mtDNA subclades II-1, 2, and 3) is roughly 34,000 years ago (estimated based on average of pairwise genetic distances assuming a substitution rate of 1.455% per site per million years, the estimated rate for the COI gene of ants; Resende et al., 2010), which apparently predates potential human dispersal. However, this divergence, along with the deep divergence between Clades I and II, may stem from accelerated mtDNA substitution rates due to recurrent *Wolbachia* sweeps (Shoemaker et al., 2000), with the assumption that the former had infected ancestor at some point in time in the past. A likely explanation for the specific genetic patterns observed in *P. longicornis* is the ant has experienced multiple human-mediated dispersal events from genetically distinct source populations followed by global dispersal (i.e., high propagule pressure as a result of rampant migration/movement). The genetic patterns we describe are similar to those found in several other globally distributed insects, especially those that are common in human-modified landscapes [e.g., German cockroach (*Blattella germanica*), American cockroach (*Periplaneta americana*); Vargo et al., 2014; von Beeren et al., 2015], further highlighting the role of human-mediated dispersal in shaping population structure of insect species closely associated with humans.

Reduction in genetic variation as a result of a population bottleneck is a common feature observed in the introduced ranges of numerous invasive species (Nei et al., 1975; Allendorf and Lundquist, 2003; Dlugosch and Parker, 2008). The general consensus is that *P. longicornis* originated in the Old World tropics, but to narrow this down further to a specific sub-region has been somewhat controversial (Wetterer, 2008). We did not find evidence for reduced mtDNA diversity in any sampled subregions of the Old World. Also, mtDNA structure within Clade II appears to be less associated with *Wolbachia* infections, and inferring the origin of this ant using mtDNA patterns of variation in Clade II remains challenging primarily due to insufficient sampling in certain areas (**Table S11**). Identification of the native range of *P. longicornis* on a finer geographic scale is further obscured by presumed frequent human-mediated dispersals, a multi-century old invasion history, and the potential effects of *Wolbachia* infections on mtDNA variation. However, it is interesting to note that haplotypes from the northern part of India and Nepal (Himalayan region) (Hap31 and Hap37) are divergent from other haplotypes in Clade I and form a clade sister to all other haplotypes in this clade (**Figure 2**), implying the populations in Himalayan region might be the source of invasive populations of Clade I. More comprehensive sampling and additional nuclear data from queens and males may help efforts to identify the likely origin and to reconstruct with more confidence the invasion history of this ant.

The loss of *Wolbachia* in invasive ranges is common, but not universal, in invasive insects possibly due to founder effects or altered selection pressures in the new habitats (Shoemaker et al., 2000; Tsutsui et al., 2003; Reuter et al., 2004; Yang et al., 2010; Nguyen et al., 2016). For example, both the red imported fire ant, *Solenopsis invicta*, and the Argentine ant, *Linepithema humile*, had higher *Wolbachia* infection prevalences in their native populations compared with introduced populations where the symbionts are nearly absent (Shoemaker et al., 2000; Tsutsui et al., 2003; Reuter et al., 2004; Yang et al., 2010). However, we did not observe a similar phenomenon in *P. longicornis*. Both *w*LonA and *w*LonF are found throughout geographic range of *P. longicornis*, including known invasive areas such as North America and Caribbean (**Figure S2**). Nevertheless, a few individuals in clade I (*w*LonA lost) and subclade II-3 (*w*LonF lost) have lost *Wolbachia* and this loss appears to be stochastic. The loss of *Wolbachia* likely is attributable to imperfect maternal transmission or natural curing events (Stevens and Wicklow, 1992; Hoffmann and Turelli, 1997; Clancy and Hoffmann, 1998).

Our simulation results suggest *w*LonA was acquired by the common ancestor of Clade I, and the fitness advantage associated with harboring *w*LonA infections compared with uninfected ants may have facilitated the spread of *Wolbachia* and the associated mtDNA haplotype. One possible fitness advantage of harboring *w*LonA infections could be *Wolbachia*-induced cytoplasmic incompatibility (CI). Although spread of *Wolbachia* inducing CI in haplodiploid species appears to be less efficient than in diploid species (Vavre et al., 2000), limited movement of *P. lonigicornis* (Trager, 1984; Harris and Berry, 2005) could have enabled *Wolbachia* to increase in frequency within small local populations through genetic drift, allowing the bacterium to exceed the threshold frequency for spread in a host population (Vavre et al., 2003). While this possibility remains to be tested, a survey of *Wolbachia* prevalence across numerous ant species appears supportive that *Wolbachia* infections generally are more prevalent in ant species that have limited mobility (i.e. reproducing by budding or fusion) (Wenseleers et al., 1998).

In contrast, *w*LonF appears to have been gained and lost multiple times in *P*. *longicornis* over evolutionary time and has had little or no significant effect on host mtDNA variation. One possible explanation for this pattern is that *w*LonF is simply a passive passenger in longhorn crazy ant (e.g., having negligible fitness effects), and persists because the rate of *w*LonF loss occurs roughly at the same rate as horizontal transmission (Hoffmann et al., 1996; Charlat et al., 2004; Bouwma and Shoemaker, 2011). Invasive species may acquire new *Wolbachia* in their new environments (Rocha et al., 2005; Himler et al., 2011; Schuler et al., 2013). For example, the North American fruit fly, *Rhagoletis cingulata* (Diptera: Tephritidae) acquired a new *Wolbachia* strain through interspecific horizontal transmission from the Eurasian endemic *R. cerasi* (Schuler et al., 2013). However, supergroup F *Wolbachia*, to the best of our knowledge, has only been discovered in a single ant species, *Ocymyrmex picardi* (Russell et al., 2009), suggesting that the prevalence of this variant is very low in ants, and that acquisition of this variant from other sympatric ant species in the introduced range of *P. longicornis* appears unlikely. Effective and efficient horizontal transmission of *Wolbachia* depends on intimate ecological associations that provide opportunities to bring *Wolbachia* into close contact with novel hosts (Sintupachee et al., 2006; Stahlhut et al., 2010; Boivin et al., 2014). Host-parasitoid associations have been commonly suggested as a route of the *Wolbachia* horizontal transmission, and evidence this has occurred includes between host and parasitoid (Heath et al., 1999), between hosts (Ahmed et al., 2015), and between parasitoids sharing the same host (Huigens et al., 2000; Huigens et al., 2004). Other putative ecological associations for successful *Wolbachia* horizontal transmission are prey-predator and parasite-host associations (Le Clec’h et al., 2013; Brown and Lloyd, 2015). One well-known case for the latter involves an inquiline social parasite (*Solenopsis daguerrei*) and its ant host (*S. invicta*) (Dedeine et al., 2005). While no social parasites have been reported in *P. longicornis* to date, colonies of this ant often host a variety of arthropods, such as the ant cricket *Myrmecophilus americanus*, the beetle *Coluocera maderae* and the ant mite *Macrodinychus multispinosus* (Wollaston, 1854; Wetterer, 2008; Werren et al., 2008; Lachaud et al., 2016). These arthropods, termed “myrmecophiles,” represent good candidates for *Wolbachia* transfer because they all have intimate ecological associations and interactions with their ant hosts (Kronauer and Pierce, 2011). A future study of *Wolbachia* in the organisms ecologically associated with *P. longicornis* may uncover the routes and mechanisms underlying *Wolbachia* horizontal transmission in this ant.

## Data Availability

MtDNA sequences (accession numbers KY769964-KY770018) are deposited at GenBank. The sequences of the ﬁve MLST genes and *wsp* gene of two *Wolbachia* strains are deposited at PubMLST database (ID:1827 and 1828).

## Ethics Statement

We thank Future Development Funding Program of the Kyoto University Research Coordination Alliance for financial support.

## Author Contributions

CCSY, S-PT, and DS originally conceived the ideas. JW, AS, and C-YL helped develop an overarching research programme and collect the samples. TY and CCSY applied the grant. SPT performed the experiments. S-PT, DS, and CCSY carried out the analyses and drafted the manuscript. All authors contributed substantially to editing the manuscript.

## Funding

We thank Future Development Funding Program of the Kyoto University Research Coordination Alliance for financial support.

## Conflict of Interest Statement

The authors declare that the research was conducted in the absence of any commercial or financial relationships that could be construed as a potential conflict of interest.
