# Supplementary material for: Genetic Diversity and Wolbachia Infection Patterns in a Globally Distributed Invasive Ant
Source: Front Genet. 2019 Sep 17;10:838. doi: 10.3389/fgene.2019.00838 (PMC6758599; doi:10.3389/fgene.2019.00838)
Supplement: Supplementary file 1 [file DataSheet_1.docx]

**Supporting tables**

**Table S1** Profiles for *Paratrechina longicornis* specimens in this study.

| ID | mt Clade | Haplotype | Infection type | Latitude | Longitude | Region | Country | nDNA |
| --- | --- | --- | --- | --- | --- | --- | --- | --- |
| plJP01 | I | Hap15 | A | 26.21891 | 127.68644 | Northeast Asia | Japan | Y |
| plJP02-2 | II-3 | Hap11 | F | 26.1784797 | 127.7994308 | Northeast Asia | Japan | Y |
| plJP03 | II-3 | Hap23 | F | 24.35555 | 124.24238 | Northeast Asia | Japan | Y |
| plJP04 | I | Hap15 | AF | 24.39036333 | 124.2460911 | Northeast Asia | Japan | Y |
| plJP05 | I | Hap33 | A | 24.31386 | 123.90633 | Northeast Asia | Japan | Y |
| plJP06 | I | Hap8 | N | 24.33107972 | 123.9090897 | Northeast Asia | Japan | Y |
| plJP07 | II-3 | Hap23 | F | 26.398756 | 127.758078 | Northeast Asia | Japan | Y |
| plJP08 | I | Hap8 | N | 26.333547 | 127.787008 | Northeast Asia | Japan | Y |
| plJP09 | II-3 | Hap11 | F | 26.167364 | 127.828897 | Northeast Asia | Japan | Y |
| plJP10 | I | Hap15 | AF | 26.14476 | 127.66473 | Northeast Asia | Japan | Y |
| plJP11 | I | Hap15 | A | 26.0957 | 127.6828 | Northeast Asia | Japan | Y |
| plJP12 | I | Hap21 | AF | 26.096 | 127.7218 | Northeast Asia | Japan | Y |
| plJP13 | I | Hap8 | N | 26.14186 | 127.74891 | Northeast Asia | Japan | Y |
| plJP14 | I | Hap8 | N | 26.13774 | 127.72902 | Northeast Asia | Japan | Y |
| plJP15 | II-3 | Hap11 | F | 26.6777 | 127.8912 | Northeast Asia | Japan | Y |
| plJP16 | II-3 | Hap11 | F | 26.33846 | 127.84567 | Northeast Asia | Japan | Y |
| plJP17 | I | Hap9 | AF | 26.36461 | 127.85358 | Northeast Asia | Japan | Y |
| plJP18 | I | Hap15 | AF | 26.23266 | 127.68115 | Northeast Asia | Japan | Y |
| plJP19 | II-2 | Hap19 | N | 26.23752 | 127.67418 | Northeast Asia | Japan | Y |
| plJP20 | II-2 | Hap19 | N | 26.24146 | 127.6792 | Northeast Asia | Japan | Y |
| plJP21 | I | Hap8 | N | 26.43612 | 127.79304 | Northeast Asia | Japan | Y |
| plJP22 | I | Hap8 | N | 26.20983 | 127.65172 | Northeast Asia | Japan | Y |
| pl01-4 | I | Hap8 | N | 25.157405 | 121.401672 | East Asia | Taiwan | Y |
| pl02-2 | I | Hap15 | AF | 25.056328 | 121.224689 | East Asia | Taiwan | Y |
| pl03-2 | II-1 | Hap2 | F | 24.828056 | 121.071289 | East Asia | Taiwan | Y |
| pl04 | I | Hap8 | N | 24.81848 | 121.12813 | East Asia | Taiwan | Y |
| pl05 | I | Hap8 | A | 24.81684 | 121.11055 | East Asia | Taiwan | Y |
| pl06 | II-2 | Hap1 | N | 24.49518 | 120.82783 | East Asia | Taiwan | Y |
| pl07 | I | Hap21 | AF | 24.06126 | 120.43017 | East Asia | Taiwan | Y |
| pl08 | II-1 | Hap2 | F | 24.98925 | 121.46495 | East Asia | Taiwan | Y |
| pl09 | II-3 | Hap25 | F | 25.05575 | 121.1943 | East Asia | Taiwan |  |
| pl10 | I | Hap8 | A | 23.96461 | 120.57375 | East Asia | Taiwan |  |
| pl11 | I | Hap9 | A | 24.08009 | 120.55845 | East Asia | Taiwan |  |
| pl114 | I | Hap9 | AF | 22.66592 | 120.31297 | East Asia | Taiwan |  |
| pl115 | II-1 | Hap10 | N | 22.62255 | 120.28884 | East Asia | Taiwan |  |
| pl12 | I | Hap9 | A | 24.08269 | 120.55847 | East Asia | Taiwan |  |
| pl13 | I | Hap9 | A | 24.0808 | 120.5587 | East Asia | Taiwan |  |
| pl14 | II-2 | Hap1 | N | 23.97484 | 120.68483 | East Asia | Taiwan |  |
| pl15 | I | Hap15 | A | 24.07217 | 120.87296 | East Asia | Taiwan | Y |
| pl17 | I | Hap8 | A | 23.75804 | 120.6715 | East Asia | Taiwan |  |
| pl171 | I | Hap8 | A | 22.6258 | 120.341 | East Asia | Taiwan | Y |
| pl172 | II-1 | Hap2 | F | 22.62573 | 120.36373 | East Asia | Taiwan | Y |
| pl18 | I | Hap15 | AF | 23.80967 | 120.72036 | East Asia | Taiwan |  |
| pl180 | I | Hap16 | AF | 22.75782 | 121.10272 | East Asia | Taiwan |  |
| pl182 | II-1 | Hap2 | F | 22.77547 | 121.1477 | East Asia | Taiwan |  |
| pl183 | I | Hap8 | A | 22.53192 | 120.96728 | East Asia | Taiwan | Y |
| pl185 | II-2 | Hap17 | N | 23.56713 | 119.56514 | East Asia | Taiwan |  |
| pl186 | I | Hap8 | N | 23.5676 | 119.5621 | East Asia | Taiwan |  |
| pl187 | I | Hap9 | A | 23.55607 | 119.6021 | East Asia | Taiwan |  |
| pl189 | II-3 | Hap11 | F | 23.66052 | 119.56007 | East Asia | Taiwan | Y |
| pl19 | I | Hap8 | AF | 23.91944 | 120.67461 | East Asia | Taiwan |  |
| pl195 | I | Hap18 | AF | 22.06374 | 121.56617 | East Asia | Taiwan | Y |
| pl200 | I | Hap9 | A | 23.56369 | 119.48947 | East Asia | Taiwan | Y |
| pl208 | I | Hap8 | AF | 24.22642 | 120.8794 | East Asia | Taiwan | Y |
| pl21 | I | Hap8 | N | 23.76022 | 120.61775 | East Asia | Taiwan | Y |
| pl22 | II-1 | Hap2 | F | 23.48456 | 120.468 | East Asia | Taiwan |  |
| pl225 | II-1 | Hap2 | F | 22.91977 | 121.13971 | East Asia | Taiwan | Y |
| pl23 | II-2 | Hap19 | N | 23.11964 | 120.36213 | East Asia | Taiwan | Y |
| pl230 | I | Hap9 | AF | 21.93197 | 120.82416 | East Asia | Taiwan | Y |
| pl233 | I | Hap20 | AF | 22.0025 | 120.7456 | East Asia | Taiwan | Y |
| pl24 | I | Hap9 | A | 23.14064 | 120.32557 | East Asia | Taiwan |  |
| pl26 | I | Hap21 | A | 23.13657 | 120.30029 | East Asia | Taiwan | Y |
| pl27 | II-1 | Hap2 | F | 23.29165 | 120.39574 | East Asia | Taiwan |  |
| pl29 | II-1 | Hap10 | N | 22.65576 | 120.29132 | East Asia | Taiwan |  |
| pl30 | II-1 | Hap2 | N | 22.67611 | 120.3119 | East Asia | Taiwan |  |
| pl31 | II-2 | Hap1 | N | 22.64306 | 120.6106 | East Asia | Taiwan |  |
| pl32 | I | Hap21 | A | 22.59472 | 120.61087 | East Asia | Taiwan |  |
| pl33 | I | Hap8 | AF | 24.9022 | 121.8621 | East Asia | Taiwan | Y |
| pl35 | I | Hap21 | A | 23.78891 | 120.47762 | East Asia | Taiwan |  |
| pl36 | I | Hap22 | AF | 23.8253 | 120.4559 | East Asia | Taiwan |  |
| pl37 | II-1 | Hap2 | F | 23.79779 | 120.46528 | East Asia | Taiwan | Y |
| pl38 | II-1 | Hap2 | F | 23.79232 | 120.44801 | East Asia | Taiwan |  |
| pl39 | I | Hap8 | AF | 23.77125 | 120.41276 | East Asia | Taiwan |  |
| pl40 | II-1 | Hap2 | N | 23.76207 | 120.38989 | East Asia | Taiwan |  |
| pl41 | I | Hap8 | N | 23.76186 | 120.359 | East Asia | Taiwan |  |
| pl42 | I | Hap8 | AF | 23.65795 | 120.31242 | East Asia | Taiwan |  |
| pl43 | I | Hap8 | N | 23.61172 | 120.30735 | East Asia | Taiwan |  |
| pl44 | II-1 | Hap2 | F | 23.552 | 120.3471 | East Asia | Taiwan |  |
| pl45 | I | Hap9 | A | 23.45888 | 120.3325 | East Asia | Taiwan | Y |
| pl46 | I | Hap21 | AF | 23.46512 | 120.24691 | East Asia | Taiwan | Y |
| pl47 | II-2 | Hap19 | N | 23.41133 | 120.30818 | East Asia | Taiwan |  |
| pl48 | I | Hap8 | AF | 23.4287 | 120.3979 | East Asia | Taiwan |  |
| pl49 | I | Hap8 | AF | 22.99459 | 120.23331 | East Asia | Taiwan |  |
| pl50 | II-2 | Hap1 | N | 22.99847 | 120.19744 | East Asia | Taiwan |  |
| pl51 | II-2 | Hap1 | N | 22.99391 | 120.20748 | East Asia | Taiwan |  |
| pl58 | II-3 | Hap11 | F | 22.52346 | 120.46428 | East Asia | Taiwan |  |
| pl69 | II-3 | Hap23 | F | 23.899 | 121.5503 | East Asia | Taiwan | Y |
| pl74 | II-2 | Hap1 | N | 23.96713 | 121.60876 | East Asia | Taiwan |  |
| pl76 | I | Hap24 | A | 24.02891 | 121.62731 | East Asia | Taiwan |  |
| plCN01 | I | Hap26 | A | 21.39219 | 101.31512 | East Asia | China | Y |
| plCN02 | I | Hap21 | AF | 22.29695007 | 114.1742821 | East Asia | China | Y |
| plCN04.2 | I | Hap8 | AF | 22.25664636 | 113.9027274 | East Asia | China | Y |
| plCN07 | II-1 | Hap27 | N | 22.27944 | 114.1579 | East Asia | China | Y |
| plCN09 | I | Hap28 | A | 22.26393 | 114.23711 | East Asia | China | Y |
| plCN10 | II-3 | Hap11 | N | 22.29715 | 114.27222 | East Asia | China | Y |
| plCN11 | II-2 | Hap19 | N | 22.19617 | 113.54118 | East Asia | China | Y |
| plCN12 | I | Hap8 | N | 22.50213 | 114.12656 | East Asia | China | Y |
| plCN13 | I | Hap8 | AF | 22.41307 | 114.21012 | East Asia | China | Y |
| plCN14 | II-1 | Hap2 | F | 22.31048 | 114.1581 | East Asia | China | Y |
| plCN15 | II-2 | Hap3 | N | 22.28171 | 114.18877 | East Asia | China | Y |
| plCN16 | II-3 | Hap29 | F | 22.53706 | 114.05406 | East Asia | China | Y |
| plCN17 | II-3 | Hap4 | F | 22.54667 | 114.12677 | East Asia | China | Y |
| plCN18 | II-3 | Hap29 | F | 22.54469 | 114.08518 | East Asia | China | Y |
| pl12.348 | II-3 | Hap11 | F | 21.683 | 102.1 | South Asia | Laos | Y |
| plID01 | II-1 | Hap2 | F | -0.828076 | 100.53021 | South Asia | Indonesia | Y |
| plID02 | II-2 | Hap19 | N | -8.50957 | 115.261704 | South Asia | Indonesia | Y |
| plKH02 | II-1 | Hap2 | F | 13.412693 | 103.867024 | South Asia | Cambodia | Y |
| plKH03 | II-2 | Hap1 | N | 13.369017 | 103.864459 | South Asia | Cambodia | Y |
| plMY01 | I | Hap8 | AF | 3.21726 | 101.72442 | South Asia | Malaysia | Y |
| plMY02 | I | Hap34 | AF | 5.661726 | 100.508539 | South Asia | Malaysia | Y |
| plMY03 | I | Hap8 | AF | 5.612921 | 100.486052 | South Asia | Malaysia | Y |
| plMY04 | I | Hap34 | AF | 5.41792 | 100.337 | South Asia | Malaysia | Y |
| plMY05 | II-2 | Hap19 | N | 5.325918 | 100.287427 | South Asia | Malaysia | Y |
| plMY07 | II-2 | Hap19 | N | 5.277888 | 100.27023 | South Asia | Malaysia | Y |
| plMY08 | I | Hap21 | AF | 5.372663 | 100.237777 | South Asia | Malaysia | Y |
| plMY09 | II-1 | Hap27 | N | 5.406769 | 100.280058 | South Asia | Malaysia | Y |
| plMY10 | II-1 | Hap2 | F | 5.440082 | 100.287224 | South Asia | Malaysia | Y |
| plMY101 | I | Hap21 | AF | 3.42173 | 115.152667 | South Asia | Malaysia | Y |
| plMY11 | I | Hap21 | AF | 5.354718 | 100.300785 | South Asia | Malaysia | Y |
| plMY13 | I | Hap8 | AF | 1.863265 | 102.966965 | South Asia | Malaysia | Y |
| plMY14 | II-1 | Hap10 | N | 1.841309 | 102.955261 | South Asia | Malaysia | Y |
| plMY15 | I | Hap21 | A | 1.842982 | 102.93743 | South Asia | Malaysia | Y |
| plMY16 | II-2 | Hap19 | N | 1.858027 | 102.940938 | South Asia | Malaysia | Y |
| plMY17 | II-2 | Hap19 | N | 5.661996 | 100.502915 | South Asia | Malaysia | Y |
| plMY18 | II-1 | Hap27 | N | 5.674444 | 100.508889 | South Asia | Malaysia | Y |
| plMY19 | II-2 | Hap3 | N | 5.62605 | 100.46523 | South Asia | Malaysia | Y |
| plMY20 | I | Hap8 | A | 1.486775 | 103.930877 | South Asia | Malaysia | Y |
| plMY21 | I | Hap15 | A | 1.487547 | 103.929632 | South Asia | Malaysia | Y |
| plMY22 | II-1 | Hap10 | N | 1.492245 | 103.927948 | South Asia | Malaysia | Y |
| plMY23 | I | Hap8 | AF | 1.494658 | 103.922013 | South Asia | Malaysia | Y |
| plMY24 | I | Hap21 | AF | 2.137032 | 102.505825 | South Asia | Malaysia | Y |
| plMY25 | II-1 | Hap10 | N | 1.871323 | 102.996996 | South Asia | Malaysia | Y |
| plMY26 | II-2 | Hap19 | N | 5.601889 | 100.480544 | South Asia | Malaysia | Y |
| plMY27 | I | Hap21 | A | 5.369258 | 100.248221 | South Asia | Malaysia | Y |
| plMY28 | I | Hap15 | F | 1.411972 | 103.845186 | South Asia | Singapore | Y |
| plMY29 | II-1 | Hap35 | N | 1.275823 | 103.624204 | South Asia | Singapore | Y |
| plMY30 | II-3 | Hap36 | N | 1.312363 | 103.939681 | South Asia | Singapore | Y |
| plMY31 | II-1 | Hap10 | N | 1.335399 | 103.745502 | South Asia | Singapore | Y |
| plMY32 | I | Hap21 | AF | 1.294067 | 103.85398 | South Asia | Singapore | Y |
| plMY34 | II-2 | Hap3 | N | 1.338513 | 103.743131 | South Asia | Singapore | Y |
| plPH03 | I | Hap15 | AF | 6.692472 | 125.350278 | South Asia | Philippines | Y |
| plPH04-1 | II-1 | Hap2 | F | 14.156441 | 121.233857 | South Asia | Philippines | Y |
| plPH04-2 | II-1 | Hap2 | F | 14.156441 | 121.233857 | South Asia | Philippines | Y |
| plTH01 | II-2 | Hap19 | N | 13.8421 | 100.573 | South Asia | Thailand | Y |
| plTH02 | I | Hap38 | AF | 13.84194 | 100.57374 | South Asia | Thailand | Y |
| plTH03 | I | Hap39 | AF | 13.72999 | 100.53828 | South Asia | Thailand | Y |
| plTH11 | II-1 | Hap27 | N | 13.80283 | 100.55336 | South Asia | Thailand | Y |
| plTH12 | I | Hap15 | AF | 13.80625 | 100.55512 | South Asia | Thailand | Y |
| plTH16 | II-1 | Hap27 | N | 13.77629 | 100.45629 | South Asia | Thailand | Y |
| plTH17 | II-2 | Hap3 | N | 13.80057 | 100.18766 | South Asia | Thailand | Y |
| plTH18 | II-2 | Hap19 | N | 13.80889 | 100.16084 | South Asia | Thailand | Y |
| plTH19 | II-1 | Hap40 | N | 13.96531 | 100.08373 | South Asia | Thailand | Y |
| plTH20 | II-2 | Hap19 | N | 14.45797 | 100.53773 | South Asia | Thailand | Y |
| plTH21 | I | Hap15 | F | 14.593 | 100.3782 | South Asia | Thailand | Y |
| plTH23 | II-2 | Hap1 | N | 14.59216 | 100.37907 | South Asia | Thailand | Y |
| plTH24 | I | Hap8 | F | 14.54583 | 100.50112 | South Asia | Thailand | Y |
| plTH25 | I | Hap8 | AF | 14.34815 | 100.5806 | South Asia | Thailand | Y |
| plTH26 | I | Hap15 | F | 14.34539 | 100.59335 | South Asia | Thailand | Y |
| plTH27 | I | Hap15 | AF | 14.35998 | 100.59255 | South Asia | Thailand | Y |
| plTH28 | I | Hap15 | F | 14.3501 | 100.5424 | South Asia | Thailand | Y |
| plTH29 | I | Hap9 | A | 14.35295 | 100.53183 | South Asia | Thailand | Y |
| plTH30 | I | Hap15 | AF | 13.68233 | 100.65976 | South Asia | Thailand | Y |
| plTH31 | II-2 | Hap1 | N | 13.68005 | 100.66018 | South Asia | Thailand | Y |
| plTH32 | II-1 | Hap27 | N | 14.22814 | 100.70685 | South Asia | Thailand | Y |
| plTH33 | I | Hap9 | F | 14.58983 | 101.02333 | South Asia | Thailand | Y |
| plTH34 | I | Hap41 | AF | 14.83454 | 101.54985 | South Asia | Thailand | Y |
| plTH35 | II-2 | Hap3 | N | 14.8746 | 101.7244 | South Asia | Thailand | Y |
| plTH36 | I | Hap15 | AF | 15.21134 | 101.76631 | South Asia | Thailand | Y |
| plTH37 | I | Hap8 | AF | 15.29943 | 101.73737 | South Asia | Thailand | Y |
| plTH38 | I | Hap8 | AF | 14.56549 | 101.97845 | South Asia | Thailand | Y |
| plTH39 | II-2 | Hap3 | N | 14.51466 | 101.95918 | South Asia | Thailand | Y |
| plTH40 | I | Hap15 | AF | 14.46594 | 101.90431 | South Asia | Thailand | Y |
| plVN01 | I | Hap15 | F | 20.99266 | 105.49518 | South Asia | Vietnam | Y |
| plVN02-1 | II-2 | Hap43 | N | 20.99266 | 105.49518 | South Asia | Vietnam | Y |
| pl12.101 | I | Hap8 | N | 25.327 | 55.391 | Arabia | Arabia |  |
| pl12.99 | I | Hap8 | N | 25.276 | 55.3 | Arabia | Arabia |  |
| pl05.246 | II-2 | Hap3 | N | 18.357 | -65.027 | Caribbean | USA-Virgin Islands |  |
| pl05.324 | II-2 | Hap1 | F | 18.338 | -64.666 | Caribbean | USA-Virgin Islands |  |
| pl06.134 | II-2 | Hap1 | F | 18.011 | -63.043 | Caribbean | France-St Martin |  |
| pl06.264 | II-3 | Hap4 | F | 18.083 | -67.939 | Caribbean | USA-Puerto Rico |  |
| pl06.647 | II-3 | Hap5 | F | 13.364 | -61.136 | Caribbean | St. Vincent & The Grenadines |  |
| pl06.816 | II-2 | Hap1 | N | 13.761 | -60.932 | Caribbean | Saint Lucia |  |
| pl07.163 | II-3 | Hap4 | F | 17.128 | -62.612 | Caribbean | St Kitts & Nevis |  |
| pl07.382 | II-3 | Hap4 | F | 18.043 | -63.117 | Caribbean | France-St Martin |  |
| pl07.561 | II-3 | Hap6 | F | 16.79 | -62.211 | Caribbean | UK-Montserrat |  |
| pl07.681 | I | Hap7 | A | 16.772 | -62.219 | Caribbean | UK-Montserrat |  |
| pl10.62 | II-3 | Hap5 | F | 24.981 | -77.46 | Caribbean | Bahamas |  |
| pl11.24 | II-2 | Hap3 | N | 16.225 | -61.531 | Caribbean | France-Guadeloupe |  |
| pl13.382 | II-2 | Hap3 | N | 17.702 | -64.785 | Caribbean | USA-Virgin Islands |  |
| pl14.136 | II-3 | Hap14 | F | 13.117 | -59.6 | Caribbean | Barbados |  |
| plHug087 | II-2 | Hap1 | N | 14.75777 | -60.922011 | Caribbean | France-Martinique |  |
| plHug101 | II-2 | Hap3 | N | 14.683965 | -60.940304 | Caribbean | France-Martinique |  |
| plHug88 | II-3 | Hap5 | F | 14.75547 | -60.910702 | Caribbean | France-Martinique |  |
| plJM02-3 | I | Hap32 | AF | 18.32025 | -78.09965 | Caribbean | Jamaica |  |
| plJM03-3 | I | Hap8 | AF | 18.237484 | -77.050172 | Caribbean | Jamaica |  |
| plIN01-1 | II-3 | Hap11 | F | 29.386128 | 79.110206 | Indian Subcontinent | India |  |
| plIN01-2 | II-3 | Hap11 | F | 29.386128 | 79.110206 | Indian Subcontinent | India |  |
| plIN02-1 | I | Hap8 | A | 28.5411067 | 77.2107594 | Indian Subcontinent | India |  |
| plIN02-2 | I | Hap8 | A | 28.5411067 | 77.2107594 | Indian Subcontinent | India |  |
| plIN04-1 | II-2 | Hap1 | N | 27.174121 | 78.041145 | Indian Subcontinent | India |  |
| plIN04-2 | II-2 | Hap1 | N | 27.174121 | 78.041145 | Indian Subcontinent | India |  |
| plIN05-1 | I | Hap31 | N | 29.384286 | 79.106085 | Indian Subcontinent | India |  |
| plIN05-2 | I | Hap31 | N | 29.384286 | 79.106085 | Indian Subcontinent | India |  |
| plNP01 | I | Hap37 | F | 28.201085 | 83.945061 | Indian Subcontinent | Nepal |  |
| pl12.349 | II-3 | Hap5 | F | 26.529 | -80.056 | North America | USA-Florida |  |
| pl12.357 | I | Hap12 | A | 25.7454 | -80.1763 | North America | USA-Florida |  |
| plUS08 | II-3 | Hap5 | F | 29.6524 | -82.312 | North America | USA-Florida |  |
| plUS09 | II-3 | Hap5 | F | 29.6455 | -82.308 | North America | USA-Florida |  |
| plUS10 | II-3 | Hap5 | N | 29.6453 | -82.31 | North America | USA-Florida |  |
| plUS11 | II-3 | Hap5 | N | 29.6417 | -82.3106 | North America | USA-Florida |  |
| plUS12 | I | Hap9 | A | 29.6511 | -82.3737 | North America | USA-Florida |  |
| plUS13 | II-3 | Hap5 | F | 29.65072 | -82.3741 | North America | USA-Florida |  |
| plUS19 | I | Hap9 | AF | 32.81001 | -116.94625 | North America | USA-CA |  |
| plAU01 | I | Hap8 | AF | -16.87701 | 145.75383 | Oceania | Australia |  |
| plAU02 | II-2 | Hap1 | N | -12.170968 | 136.76458 | Oceania | Australia |  |
| plAU03 | II-2 | Hap1 | N | -11.385879 | 130.426509 | Oceania | Australia |  |
| plAU04 | II-2 | Hap1 | N | -11.385879 | 130.426509 | Oceania | Australia |  |
| plAU12 | I | Hap8 | AF | -16.9301307 | 145.7720749 | Oceania | Australia |  |
| plAU17 | I | Hap8 | N | -16.9133044 | 145.7700533 | Oceania | Australia |  |
| plAU19 | II-2 | Hap1 | N | -16.8733555 | 145.7563153 | Oceania | Australia |  |
| plFJ01 | I | Hap8 | AF | -17.772803 | 177.367195 | Oceania | Fiji |  |
| plFJ02 | I | Hap21 | A | -18.105823 | 178.39531 | Oceania | Fiji |  |
| plFJ03 | I | Hap21 | A | -18.113325 | 178.473873 | Oceania | Fiji |  |
| plFJ04 | I | Hap21 | A | -18.145858 | 178.447527 | Oceania | Fiji |  |
| plFJ05-1 | I | Hap8 | AF | -17.441404 | 177.861391 | Oceania | Fiji |  |
| plFJ05-2 | I | Hap8 | AF | -17.441404 | 177.861391 | Oceania | Fiji |  |
| plFJ06 | I | Hap9 | A | -17.449162 | 177.983061 | Oceania | Fiji |  |
| plFJ07 | II-2 | Hap3 | N | -17.79612 | 177.398636 | Oceania | Fiji |  |
| plFJ08 | I | Hap21 | A | -18.21001 | 177.711677 | Oceania | Fiji |  |
| plFJ09 | I | Hap21 | A | -18.08804 | 177.552228 | Oceania | Fiji |  |
| plUS01 | I | Hap8 | A | 21.307922 | -157.816293 | Polynesia | USA-Hawaii |  |
| plUS02 | II-2 | Hap3 | N | 21.270779 | -157.697124 | Polynesia | USA-Hawaii |  |
| plUS03 | II-1 | Hap2 | N | 19.410158 | -155.893298 | Polynesia | USA-Hawaii |  |
| plUS04 | I | Hap8 | A | 19.724453 | -155.084908 | Polynesia | USA-Hawaii |  |
| plUS05-2 | I | Hap8 | N | 19.662305 | -155.006883 | Polynesia | USA-Hawaii |  |
| plUS14 | I | Hap42 | N | 19.64074 | -155.99731 | Polynesia | USA-Hawaii |  |
| plUS15 | II-1 | Hap2 | F | 19.71444 | -155.03995 | Polynesia | USA-Hawaii |  |
| plUS16 | II-2 | Hap3 | N | 21.27906 | -157.82815 | Polynesia | USA-Hawaii |  |
| plUS17 | II-2 | Hap3 | N | 21.27898 | -157.83346 | Polynesia | USA-Hawaii |  |
| pl12.67 | II-1 | Hap13 | N | -16.534 | 28.803 | South Africa | Zimbabwe |  |
| pl10.201 | II-2 | Hap3 | N | 10.597 | -67.007 | South America | Venezuela |  |
| pl10.289 | I | Hap7 | N | 10.998 | -63.867 | South America | Venezuela |  |
| plGR01 | I | Hap30 | A | 36.44634 | 28.2257 | Southeastern Europe | Greece |  |
| plGR03 | I | Hap30 | A | 36.091819 | 28.088709 | Southeastern Europe | Greece |  |
| plST01-1 | II-1 | Hap27 | N | 0.367658 | 6.712249 | West Africa | São Tomé and Príncipe |  |
| plST01-2 | II-1 | Hap27 | N | 0.367658 | 6.712249 | West Africa | São Tomé and Príncipe |  |
| plST01-3 | II-1 | Hap27 | N | 0.367658 | 6.712249 | West Africa | São Tomé and Príncipe |  |
| plST01-4 | II-1 | Hap27 | N | 0.367658 | 6.712249 | West Africa | São Tomé and Príncipe |  |

**Table S2** Sequences of polymerase chain reaction (PCR) primers used for amplification of mtDNA gene.

| Name | Primer sequences (5’ – 3’) | Primer position | Ta (°C) | Reference |
| --- | --- | --- | --- | --- |
| C1-J-1745M-F^†^ | CCTCGAATAAATAATATAAGATTTTGAC | COI | 52 | Modified from (Degnan et al., 2004) |
| PLCOII-R2 | TTAGATTGCAGGAATTTCGTTATATCT | COI | 52 | This study |
| PLCOII-F1^†^ | ACCACGTCGTTATTCTGACTATC | COI | 52 | This study |
| C2-N-3661R | CCACAAATTTCTGAACATTGACCA | COII | 52 | (Degnan et al., 2004) |

^†^Primer pair C1-J-1745M-F/PLCOII-R2 amplified partial COI region (approximately 1151 bp), and PLCOII-F1/ C2-N-3661R amplified partial COI-tRNA-COII (approximately 917-928 bp) region

**Table S3** Sequences of polymerase chain reaction (PCR) primers used for detection of the reproductive parasites in this study.

| Species | Name | Primer sequences (5’ – 3’) | Ta (°C) | Size (bp) | Reference |
| --- | --- | --- | --- | --- | --- |
| *Wolbachia* sp. | 81F | TGGTCCAATAAGTGATGAAGAAAC | 50 | 610 | (Zhou et al., 1998) |
|  | 691R | AAAAATTAAACGCTACTCCA |  |  |  |
|  | 16SwolF^†^ | TTGTAGCCTGCTATGGTATAACT | 54 | 896 | (O’Neill et al., 1992) |
|  | 16SwolR^†^ | GAATAGGTATGATTTTCATGT |  |  |  |
|  | fts-F^†^ | GTATGCCGATTGCAGAGCTTG | 55 | 769 | (Kondo et al., 1999) |
|  | fts-R^†^ | GCCATGAGTATTCACTTGGCT |  |  |  |

^†^ Primers were used to double-check the infection status of *Wolbachia* when the result of *Wolbachia* detection by using primer 81F and 691R was negative.

**Table S4** Specific primers used for detection and amplification of *Wolbachia* in this study.

| DNA target | Name | Primer sequences (5’ – 3’) | Ta (°C) | Size (bp) | Reference |
| --- | --- | --- | --- | --- | --- |
| *Wolbachia* A supergroup | 136F | TGAAATTTTACCTCTTTTC | 50 | 556 | (Zhou et al., 1998) |
|  | 691R | AAAAATTAAACGCTACTCCA |  |  |  |
| *Wolbachia* B supergroup | 81F | TGGTCCAATAAGTGATGAAGAAAC | 50 | 442 | (Zhou et al., 1998) |
|  | 522R | ACCAGCTTTTGCTTGATA |  |  |  |
| *Wolbachia* *w*LonA | WspSpePlA-F | GTTCGTTTGCAATACAACGGTG | 54 | 430 | This study |
|  | WspSpePlA-R | TGTCATAGCTGACACCAGCTCTTGC |  |  |  |
| *Wolbachia* *w*LonF | WspSpePlF-F | AAGGTGATAAAGATCAAGATCCTT | 54 | 439 | This study |
|  | WspSpePlF-R | TACCATCACCCTTAGTTGTTGCAT |  |  |  |

**Table S5** Sequences of polymerase chain reaction (PCR) primers used for amplification of microsatellite loci.

| Name | Repeat motif | | Primer sequences (5’ – 3’) | Ta (°C) | Reference |
| --- | --- | --- | --- | --- | --- |
| Prl102 | (CT)^12 | F: | TCCAACTGACCCGGAAGAC | 58 | (Tseng et al., 2019) |
|  |  | R: | CGTACGGAATCGTGCGAAG |  |  |
| Prl104 | (AG)^15 | F: | GAGAGGGAACCCTGCTTCG | 58 | (Tseng et al., 2019) |
|  |  | R: | TCTGCCTGGTTTAGCCCTC |  |  |
| Prl106 | (AT)^17 | F: | CTCATCGACCCTTTGACGG | 58 | (Tseng et al., 2019) |
|  |  | R: | ACTGGTAAGTCCACTCCGC |  |  |
| Prl107 | (AT)^10 | F: | TCTCTGCAGCTGTGTCAGG | 58 | (Tseng et al., 2019) |
|  |  | R: | CGCAATTAGCGTCTCCGC |  |  |
| Prl109 | (CT)^12 | F: | CAGTCGCAACAATGGCGG | 58 | (Tseng et al., 2019) |
|  |  | R: | TGACGAAAGCACCCGTAGG |  |  |
| Prl110 | (CT)^15 | F: | CGTTATCCGTTCGTCACCG | 58 | (Tseng et al., 2019) |
|  |  | R: | GTGTCCGATGCAAATCCCG |  |  |
| Prl111 | (AG)^13 | F: | AGCTGTCTGATTTCGTCGC | 58 | (Tseng et al., 2019) |
|  |  | R: | AACGCCTTTAATCCGTCGC |  |  |
| Prl113 | (AT)^10 | F: | ATACACATTAGTGCATCCAACC | 58 | (Tseng et al., 2019) |
|  |  | R: | TTCGGCGTTCGTGAACAAG |  |  |
| Prl118 | (AG)^16 | F: | ACAGGAAGTCGCGGAGATG | 58 | (Tseng et al., 2019) |
|  |  | R: | AATGCGGTGGTCAAAGTGC |  |  |
| Prl119 | (AT)^13 | F: | ACAACTAATCGCCCGTAGC | 58 | (Tseng et al., 2019) |
|  |  | R: | TGGATCGTGAGATTTCCGTTTAG |  |  |
| Prl120 | (AG)^17 | F: | CGCATGTGAATGTAAACGATGG | 58 | (Tseng et al., 2019) |
|  |  | R: | CAGCTTGCGGTTCAAGGTC |  |  |
| Prl121 | (CT)^10 | F: | TAGTGCTGGATGCAGGGTG | 58 | (Tseng et al., 2019) |
|  |  | R: | ACGGCGTAGTACCTTCTGC |  |  |
| Prl123 | (AG)^12 | F: | ACCGCAGCGTTAATTGC | 58 | (Tseng et al., 2019) |
|  |  | R: | GTCTCCGGACCCATTCTCG |  |  |
| Prl125 | (CT)^10 | F: | AACACGGATGATTGCATGTC | 58 | (Tseng et al., 2019) |
|  |  | R: | GCCGTGATACGAACTTCCAC |  |  |
| Prl126 | (AT)^11 | F: | AAGAACTGCAAGAGTGCGG | 58 | (Tseng et al., 2019) |
|  |  | R: | GCACGTCCCGAGAAACATC |  |  |
| Prl127 | (AG)^12 | F: | AGCTTCCCGTACTTACACG | 58 | (Tseng et al., 2019) |
|  |  | R: | TGCAGAAAGTATGTCGCGATG |  |  |
| Prl128 | (AT)^15 | F: | AAATTCGTCATGTTCCAGATCC | 58 | (Tseng et al., 2019) |
|  |  | R: | CAGCTGGCAAGGCATGAAC |  |  |
| Prl130 | (CT)^11 | F: | GCACGCGGAAGCAATTAAC | 58 | (Tseng et al., 2019) |
|  |  | R: | GGACGCGTTGGAAAGTTCG |  |  |
| Prl132 | (CT)^14 | F: | GATGGCGGAAATACCGGAG | 58 | (Tseng et al., 2019) |
|  |  | R: | TCGTTGACTTTACGTGTCGC |  |  |
| Prl136 | (AT)^14 | F: | TTGACACAGAAGGCATTTCG | 58 | (Tseng et al., 2019) |
|  |  | R: | AGACGGGAGGAAATATCACGG |  |  |

**Table S****6** Regional genetic diversity of *Paratrechina longicornis*. Number of individuals sampled (N), number of segregating sites (*S*), number of haplotypes (*h*), haplotype diversity (*Hd*), and nucleotide diversity (*π*/bp). *w*LonA+, *w*LonA-, *w*LonF+, and *w*LonF- denote *w*LonA-infected, *w*LonA**-**uninfected, *w*LonF-infected, and *w*LonF**-**uninfected ants in a given region, respectively. Note that the *w*LonA**+** group includes *w*LonA and F co-infected ants and *w*LonA single infected ants, and *w*LonA- group includes uninfected ants and *w*LonF single infected ants.

| Geographic regions ^†^ | N | *S* | *h* | *Hd* | *π*/bp |
| --- | --- | --- | --- | --- | --- |
| All | 248 | 172 | 43 | 0.922 | 0.034 |
| *w*LonA+ | 98 | 21 | 20 | 0.818 | 0.001 |
| *w*LonA- | 150 | 164 | 27 | 0.922 | 0.026 |
| *w*LonF+ | 111 | 146 | 24 | 0.898 | 0.032 |
| *w*LonF- | 137 | 161 | 27 | 0.909 | 0.034 |
| Northeast Asia | 22 | 125 | 8 | 0.857 | 0.031 |
| *w*LonA+ | 8 | 4 | 4 | 0.643 | 0.001 |
| *w*LonA- | 14 | 122 | 4 | 0.747 | 0.035 |
| *w*LonF+ | 11 | 117 | 4 | 0.691 | 0.022 |
| *w*LonF- | 11 | 110 | 5 | 0.818 | 0.034 |
| East Asia | 81 | 137 | 23 | 0.887 | 0.033 |
| *w*LonA+ | 39 | 12 | 11 | 0.804 | 0.001 |
| *w*LonA- | 42 | 130 | 13 | 0.858 | 0.025 |
| *w*LonF+ | 38 | 124 | 14 | 0.861 | 0.033 |
| *w*LonF- | 43 | 133 | 15 | 0.878 | 0.032 |
| South Asia | 71 | 136 | 19 | 0.920 | 0.034 |
| *w*LonA+ | 28 | 7 | 8 | 0.812 | 0.001 |
| *w*LonA- | 43 | 135 | 14 | 0.906 | 0.024 |
| *w*LonF+ | 36 | 121 | 10 | 0.835 | 0.018 |
| *w*LonF- | 35 | 133 | 13 | 0.889 | 0.022 |
| Indian Subcontinent | 9 | 145 | 5 | 0.889 | 0.041 |
| *w*LonA+ | 2 | 0 | 1 | 0.000 | 0.000 |
| *w*LonA- | 7 | 137 | 4 | 0.857 | 0.041 |
| *w*LonF+ | 3 | 106 | 2 | 0.667 | 0.040 |
| *w*LonF- | 6 | 131 | 3 | 0.800 | 0.041 |
| Oceania | 17 | 115 | 5 | 0.772 | 0.028 |
| *w*LonA+ | 11 | 2 | 3 | 0.636 | 0.000 |
| *w*LonA- | 6 | 114 | 3 | 0.600 | 0.022 |
| *w*LonF+ | 5 | 0 | 1 | 0.000 | 0.000 |
| *w*LonF- | 12 | 115 | 5 | 0.758 | 0.034 |
| Polynesia | 9 | 124 | 4 | 0.806 | 0.038 |
| *w*LonA+ | 2 | 0 | 1 | 0.000 | 0.000 |
| *w*LonA- | 7 | 124 | 4 | 0.810 | 0.035 |
| *w*LonF- | 8 | 124 | 4 | 0.786 | 0.039 |
| North America | 9 | 110 | 3 | 0.556 | 0.031 |
| *w*LonA+ | 3 | 4 | 2 | 0.667 | 0.002 |
| *w*LonA- | 6 | 0 | 1 | 0.000 | 0.000 |
| *w*LonF+ | 5 | 108 | 2 | 0.400 | 0.025 |
| *w*LonF- | 4 | 110 | 3 | 0.833 | 0.042 |
| Caribbean | 19 | 128 | 9 | 0.895 | 0.023 |
| *w*LonA+ | 3 | 3 | 3 | 1.000 | 0.001 |
| *w*LonA- | 16 | 28 | 6 | 0.850 | 0.008 |
| *w*LonF+ | 12 | 125 | 7 | 0.894 | 0.023 |
| *w*LonF- | 7 | 115 | 3 | 0.667 | 0.019 |
| Arabia | 2 | 0 | 1 | 0.000 | 0.000 |
| Southeastern Europe | 2 | 0 | 1 | 0.000 | 0.000 |
| West Africa | 4 | 0 | 1 | 0.000 | 0.000 |
| South America | 2 | 115 | 2 | 1.000 | 0.066 |

^†^ South Africa excluded from analysis due to sample size of one.

**Table S7** Allelic profiles, sequence types (ST), and *wsp* allele numbers of the two *Wolbachia* strains in *Paratrechina longicornis*. The most similar allelic profiles in PubMLST database are also displayed

| Host | Strain | ID | Supergroup | ST | *gatB* | *coxA* | *hcpA* | *ftsZ* | *fbpA* | *wsp* |
| --- | --- | --- | --- | --- | --- | --- | --- | --- | --- | --- |
| *Ephestia kuehniella* | Ekue_A | 13 | A | 19 | 7 | 6 | 7 | 3 | 8 | 18 |
| *Technomyrmex albipes* | Talb_A | 111 | A | 19 | 7 | 6 | 7 | 3 | 8 | 18 |
| *Leptomyrmex* sp. | Lept_A | 115 | A | 19 | 7 | 6 | 7 | 3 | 8 | 18 |
| *Ornipholidotos peucetia* | Opeu_A | 123 | A | 19 | 7 | 6 | 7 | 3 | 8 | 18 |
| *Pheidole plagiara* | Ppla_A_20-05 | 124 | A | 19 | 7 | 6 | 7 | 3 | 8 | 18 |
| *Pheidole sauberi* | Psau_A | 125 | A | 19 | 7 | 6 | 7 | 3 | 8 | 18 |
| *Leptogenys* sp. | Lepg_A_06-03 | 146 | A | 19 | 7 | 6 | 7 | 3 | 8 | 18 |
| *Aricia artaxerxes* | Aart_A | 451 | A | 19 | 7 | 6 | 7 | 3 | 8 | 18 |
| ***P. longicornis*** | ***w*LonA** | **1827** | **A** | **19** | **7** | **6** | **7** | **3** | **8** | **18** |
| *Brachythemis contaminata* | Bcon_F_Odo3 | 360 | F | 239 | 168 | 147 | 173 | 132 | 226 | NA |
| *Orthetrum sabina* | Osab_F_Odo6 | 363 | F | 242 | 168 | 147 | 175 | 132 | 226 | NA |
| *Orthetrum sabina* | Osab_F_Odo7 | 366 | F | 243 | 168 | 147 | 177 | 132 | 226 | NA |
| ***P. longicornis*** | ***w*LonF** | **1828** | **F** | **471** | **168** | **147** | **262** | **132** | **226** | **708** |

NA, Not applicable

**Table S8** Tests for departure from neutrality for mtDNA sequence variation in *Paratrechina longicornis*. Tajima’s *D*, Fu and Li's *D**, Fu and Li's *F**, normalized Fay and Wu's *Hn*, DHEW test *P*-value, and Neutrality index from McDonald–Kreitman test (M-K test). *w*LonA+, *w*LonA-, *w*LonF+, and *w*LonF- denote *w*LonA-infected, *w*LonA**-**uninfected, *w*LonF-infected, and *w*LonF**-**uninfected ants in a given region, respectively.

| Geographic regions | N | Tajima's *D* | Fu and Li's *D** | Fu and Li's *F** | Fay and Wu's *Hn* | DHEW test *P*-value | Neutrality index |
| --- | --- | --- | --- | --- | --- | --- | --- |
| All | 248 | 2.991 | 0.839 | **2.328**** | NA | NA | 1.612 |
| *w*LonA+ | 98 | -**1.927*** | **-3.377**** | **-3.252**** | **-2.689*** | **<0.001** | **12.888***** |
| *w*LonA- | 150 | 1.445 | 1.135 | 1.550 | **-3.465**** | 0.863 | 1.014 |
| *w*LonF+ | 111 | **3.037**** | 1.087 | **2.337**** | -1.137 | 0.999 | 1.317 |
| *w*LonF- | 137 | **3.006**** | **1.615*** | **2.715**** | **-1.771*** | 0.997 | 1.481 |
| Northeast Asia | 22 | **2.249*** | **1.766**** | **2.111**** | 0.78 | 0.709 | 0.947 |
| *w*LonA+ | 8 | -0.222 | -0.176 | -0.189 | 0.780 | 0.709 | **12.737*** |
| *w*LonA- | 14 | **2.437**** | **1.699**** | **2.033**** | -0.587 | 0.353 | 0.746 |
| *w*LonF+ | 11 | -0.234 | **1.589**** | 1.155 | **-2.115*** | **0.037*** | 0.945 |
| *w*LonF- | 11 | **2.798***** | **1.623**** | **1.987**** | -0.168 | 0.193 | 0.883 |
| East Asia | 81 | **3.380***** | **1.751**** | **2.875**** | -0.801 | 1.000 | 1.131 |
| *w*LonA+ | 39 | -1.629 | **-3.082*** | **-2.877*** | **-1.920*** | **0.024*** | **9.603**** |
| *w*LonA- | 42 | 1.302 | **1.925**** | **1.915**** | **-2.82*** | 0.823 | 0.775 |
| *w*LonF+ | 38 | **3.342***** | **1.742**** | **2.630**** | -0.294 | 0.998 | 0.947 |
| *w*LonF- | 43 | **2.759**** | 1.451 | **2.257**** | -0.948 | 0.998 | 1.061 |
| South Asia | 71 | **3.454***** | **1.791**** | **2.943**** | -0.894 | 1.000 | 1.105 |
| *w*LonA+ | 28 | -0.998 | -1.462 | -1.428 | -1.828 | **0.021*** | 5.074 |
| *w*LonA- | 43 | 0.98 | **1.591*** | 1.574 | **-3.100**** | 0.707 | 1.013 |
| *w*LonF+ | 36 | 0.16 | 1.163 | 0.954 | **-3.035**** | 0.293 | 0.971 |
| *w*LonF- | 35 | 0.481 | 1.348 | 1.195 | **-3.374**** | 0.460 | 0.933 |
| Indian Subcontinent | 9 | 1.483 | **1.488*** | **1.574*** | -0.335 | 1.000 | 0.683 |
| *w*LonA- | 7 | 1.33 | 1.347 | 1.404 | -0.386 | 1.000 | 0.518 |
| *w*LonF- | 6 | 1.294 | **1.759**** | **1.691**** | -0.323 | 1.000 | 0.687 |
| Oceania | 17 | 1.984 | **1.597**** | **1.810**** | -0.888 | 0.544 | 0.839 |
| *w*LonA+ | 11 | 0.199 | -0.330 | -0.205 | 0.362 | 0.323 | 12.789 |
| *w*LonA- | 6 | **-1.546**** | **-1.587**** | **-1.584**** | **-3.454***** | **0.001**** | 0.719 |
| *w*LonF- | 12 | **2.648**** | **1.553**** | **1.910**** | -0.143 | 0.245 | 0.839 |
| Polynesia | 9 | **2.209*** | **1.627**** | **1.833**** | -0.248 | 0.089 | 0.849 |
| *w*LonA- | 7 | 1.062 | **1.667**** | **1.561*** | -1.119 | **0.028*** | 0.849 |
| *w*LonF- | 8 | **2.051*** | 1.357 | **1.574*** | -0.275 | 0.059 | 0.849 |
| North America | 9 | 1.756 | **1.585**** | **1.660**** | -1.051 | 0.095 | 0.947 |
| *w*LonF+ | 5 | -1.267 | **-1.267**** | **-1.267**** | **-3.005***** | **0.006**** | 0.768 |
| Caribbean | 19 | 0.365 | **1.540**** | 1.264 | **-3.107**** | 0.267 | 1.241 |
| *w*LonA- | 16 | **2.439*** | 1.255 | **1.673**** | 0.053 | 0.947 | 1.640 |
| *w*LonF+ | 12 | -0.313 | **1.523**** | 1.084 | -**3.133**** | 0.112 | 1.153 |
| *w*LonF- | 7 | **-1.683*** | **-1.804**** | **-1.793**** | **-3.703***** | **0.019*** | 0.839 |

**P* < 0.05; ***P* < 0.01; ****P* < 0.0001; statistics significantly deviated from expectations under neutrality
Groups with samples size smaller than 5 were excluded from the analyses

NA; Not applicable

**Table S9** Genetic diversity of *Paratrechina longicornis* in Asia regions based on 20 microsatellite markers. Sample size (N), average number of alleles (*Na*), average number of effective alleles (*Ne*), average of observed heterozygosity (*Ho*), average of expected heterozygosity (*He*), and average of Shannon’s information index (*I*)*.* *w*LonA+, *w*LonA-, *w*LonF+, and *w*LonF- denote *w*LonA-infected, *w*LonA**-**uninfected, *w*LonF-infected, and *w*LonF**-**uninfected ants in a given region, respectively.

| Geographic regions | N | *Na* | *Ne* | *Ho* | *He* | *I* |
| --- | --- | --- | --- | --- | --- | --- |
| Asia | 134 | 9.55 | 3.80 | 0.91 | 0.68 | 1.49 |
| *w*LonA+ | 56 | 7.80 | 3.53 | 0.90 | 0.66 | 1.41 |
| *w*LonA- | 78 | 8.55 | 3.87 | 0.91 | 0.69 | 1.49 |
| *w*LonF+ | 69 | 8.25 | 3.60 | 0.90 | 0.66 | 1.42 |
| *w*LonF- | 65 | 8.50 | 3.87 | 0.91 | 0.69 | 1.49 |
| Northeast Asia | 22 | 4.85 | 3.08 | 0.90 | 0.63 | 1.21 |
| *w*LonA+ | 8 | 4.30 | 3.09 | 0.89 | 0.62 | 1.18 |
| *w*LonA- | 14 | 4.00 | 2.91 | 0.91 | 0.62 | 1.13 |
| *w*LonF+ | 11 | 4.35 | 2.96 | 0.91 | 0.62 | 1.16 |
| *w*LonF- | 11 | 4.50 | 2.95 | 0.89 | 0.63 | 1.17 |
| East Asia | 41 | 7.90 | 3.76 | 0.91 | 0.68 | 1.47 |
| *w*LonA+ | 20 | 6.40 | 3.67 | 0.92 | 0.68 | 1.41 |
| *w*LonA- | 21 | 6.65 | 3.63 | 0.90 | 0.68 | 1.43 |
| *w*LonF+ | 22 | 6.75 | 3.74 | 0.90 | 0.68 | 1.44 |
| *w*LonF- | 19 | 6.25 | 3.55 | 0.92 | 0.67 | 1.38 |
| South Asia | 71 | 8.10 | 3.73 | 0.90 | 0.68 | 1.45 |
| *w*LonA+ | 28 | 6.25 | 3.16 | 0.90 | 0.64 | 1.29 |
| *w*LonA- | 43 | 7.45 | 3.91 | 0.91 | 0.69 | 1.48 |
| *w*LonF+ | 36 | 6.55 | 3.29 | 0.90 | 0.64 | 1.32 |
| *w*LonF- | 35 | 6.95 | 3.87 | 0.91 | 0.69 | 1.46 |

**Table S10** The estimated mean log probability of the data LnP(*K*), standard deviation of LnP(*K*) and *ΔK* for *Paratrechina longicornis* inferred by STRUCTURE

| *K* | Reps | Mean *LnP*(*K*) | | Stdev *LnP*(*K*) | | *Ln'(K)* | | \|*Ln*''(*K*)\| | | *ΔK* |
| --- | --- | --- | --- | --- | --- | --- | --- | --- | --- | --- |
| 1 | 5 | -7987.60 | 0.54 | | -- | | -- | | -- | |
| 2 | 5 | -7454.86 | 69.40 | | 532.74 | | 136.50 | | 1.97 | |
| 3 | 5 | -7058.62 | 11.19 | | 396.24 | | 96.40 | | 8.62 | |
| 4 | 5 | -6758.78 | 39.63 | | 299.84 | | 88.36 | | 2.23 | |
| 5 | 5 | -6547.30 | 43.26 | | 211.48 | | 95.02 | | 2.20 | |
| 6 | 5 | -6430.84 | 30.03 | | 116.46 | | 17.94 | | 0.60 | |
| 7 | 5 | -6332.32 | 90.78 | | 98.52 | | 103.60 | | 1.14 | |
| 8 | 5 | -6337.40 | 263.17 | | -5.08 | | 96.60 | | 0.37 | |
| 9 | 5 | -6245.88 | 243.52 | | 91.52 | | 1956.90 | | 8.04 | |
| 10 | 5 | -8111.26 | 3376.97 | | -1865.38 | | -- | | -- | |

**Table S11** Genetic diversity of *Paratrechina longicornis* for ants belonging to Clade II among various geographic regions. Number of individuals sampled (N), number of segregating sites (*S*), number of haplotypes (*h*), haplotype diversity (*Hd*), nucleotide diversity (*π*/bp)

| Geographic regions | N | *S* | *h* | *Hd* | *π/bp* |
| --- | --- | --- | --- | --- | --- |
| Northeast Asia | 8 | 27 | 3 | 0.714 | 0.007 |
| East Asia | 35 | 44 | 12 | 0.830 | 0.010 |
| South Asia | 36 | 49 | 11 | 0.881 | 0.009 |
| Indian Subcontinent | 4 | 23 | 2 | 0.667 | 0.009 |
| West Africa | 4 | 0 | 1 | 0.000 | 0.000 |
| Oceania | 5 | 2 | 2 | 0.400 | 0.001 |
| Polynesia | 5 | 28 | 2 | 0.600 | 0.010 |
| North America | 6 | 0 | 1 | 0.000 | 0.000 |
| Caribbean | 16 | 28 | 6 | 0.850 | 0.008 |


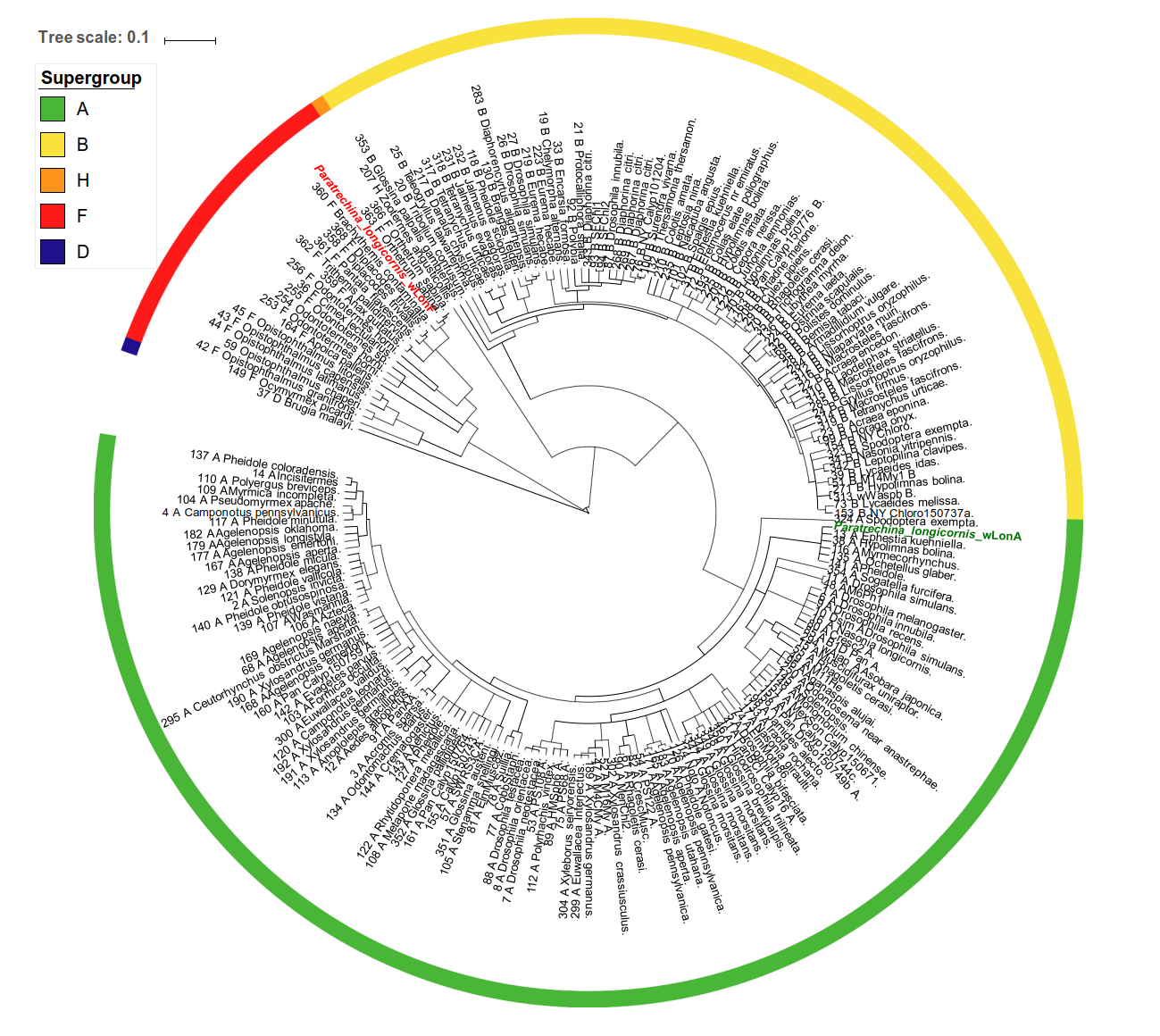


**Figure S1** CLonAlFrame genealogy of 5-locus MLST data in Genbank for *Wolbachia*. The two *Wolbachia* strains detected in this study are marked in green (*w*LonA) and red (*w*LonF). Information regarding host and *Wolbachia* supergroup is obtained from PubMLST database.


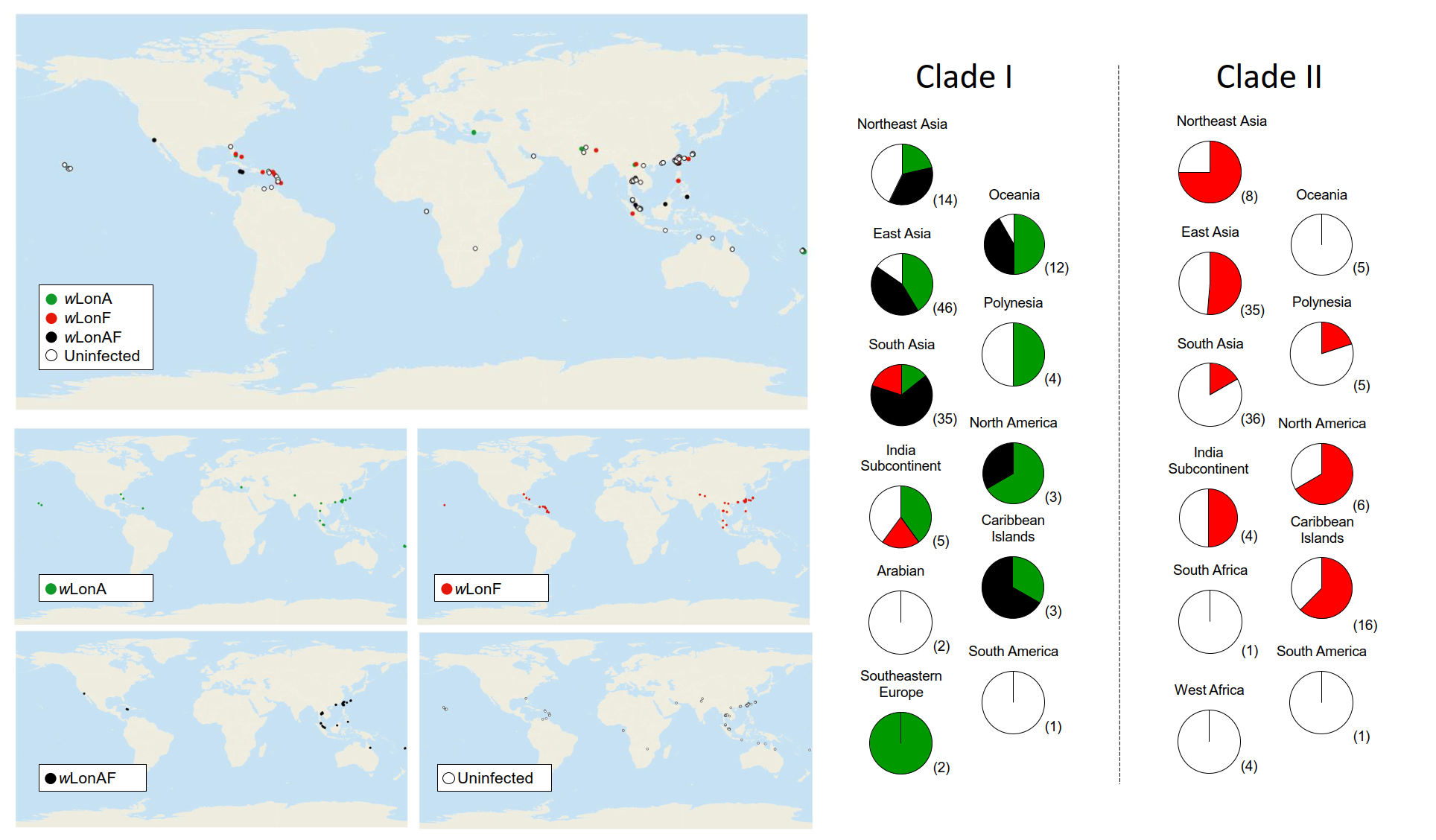


**Figure S2** Geographic distribution of *Wolbachia* infection of *Paratrechina longicornis* in the study regions. Different colors represent different infection status: *w*LonA infected (green), *w*LonF infected (red), *w*LonA and *w*LonF co-infection (black), and uninfection (white) individuals. Pie charts show the prevelance of each *Wolbachia* strain in each geographic region. Sample sizes are shown in parentheses


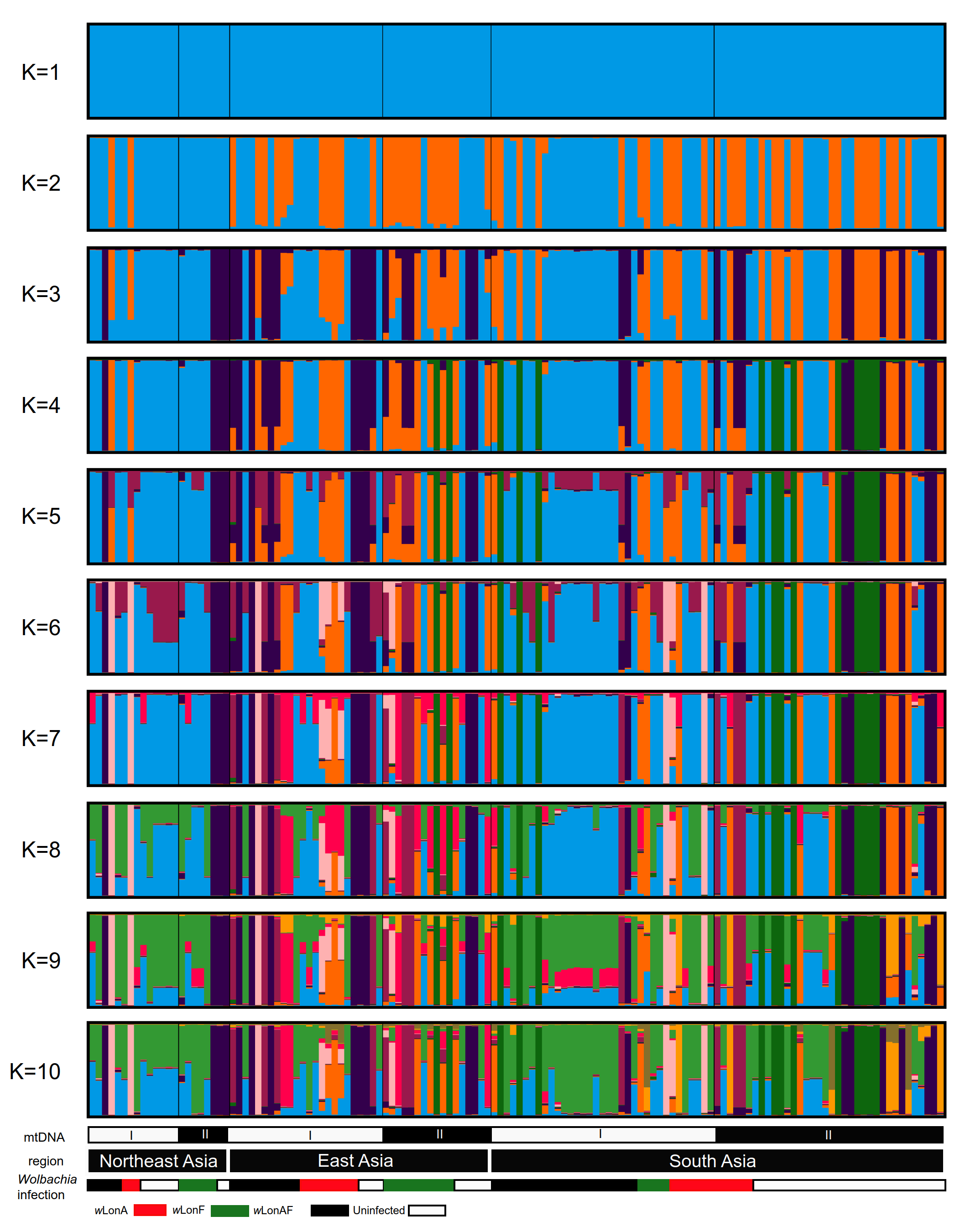


**Figure S3** *Paratrechina longicornis* population clustering analyses from Structure based on 20 microsatellite loci. Results from *K*=1 to *K*=10 are shown with the major mode generated by CLUMPAK with the highest mean posterior probability. Samples are organized by mtDNA clade, geographic regions and *Wolbachia* infection status.


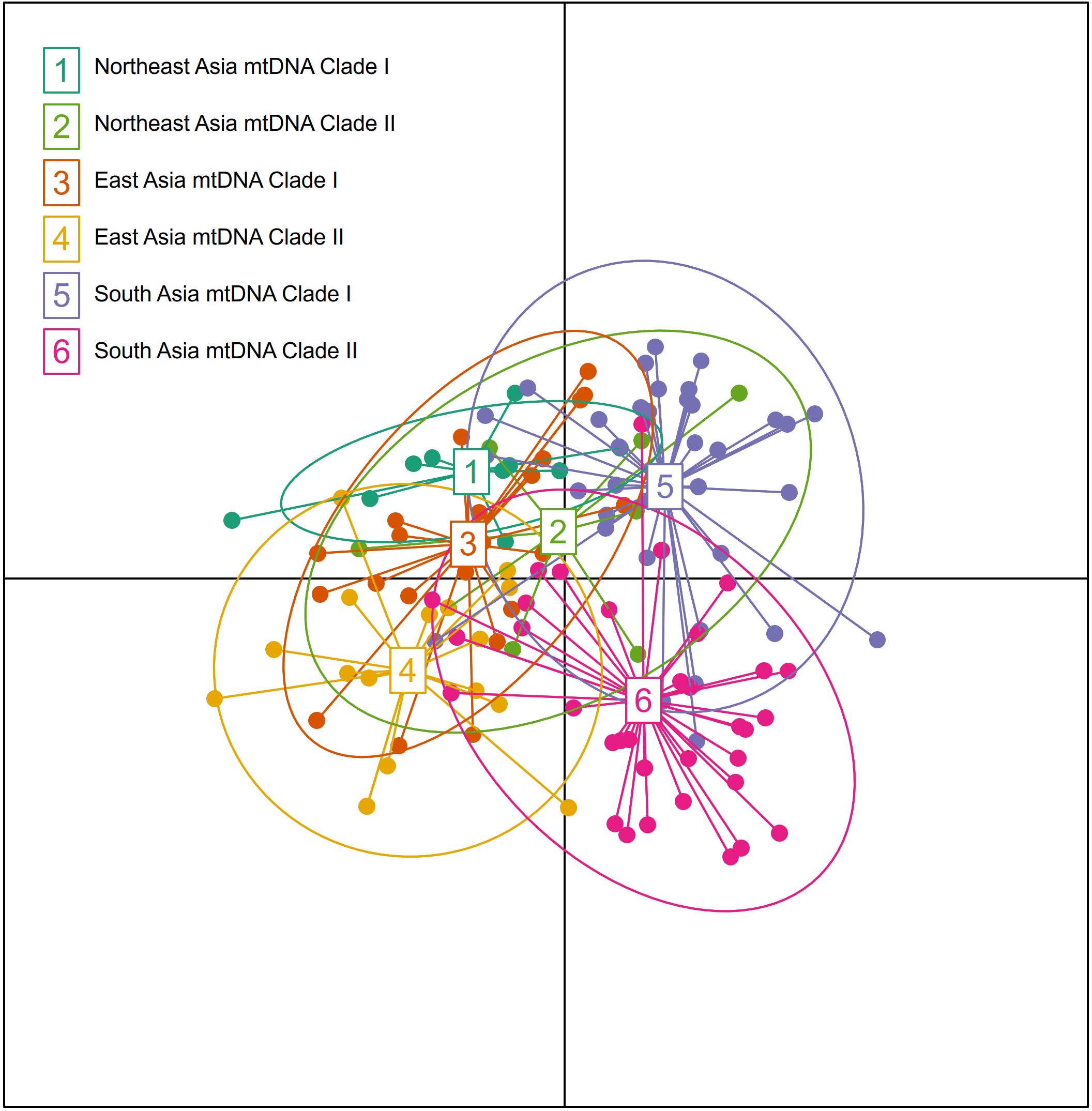


**Figure S4** Discriminant analysis of principal components (DAPC) of nuclear DNA variation for *Paratrechina longicornis* populations from selected Asia regions. Area identities are labelled in the center of the dispersion, while the large open circle indicates the 90% inertia ellipses for each group.

**Reference**

Degnan, P. H., Lazarus, A. B., Brock, C. D., and Wernegreen, J. J. (2004). Host–symbiont stability and fast evolutionary rates in an ant–bacterium association: cospeciation of *Camponotus* species and their endosymbionts, *Candidatus* Blochmannia. *Syst. Biol.* 53, 95-110.

Kondo, N., Shimada, M., and Fukatsu, T. (1999). High prevalence of *Wolbachia* in the Azuki Bean Beetle *Callosobruchus chinensis* (Coleoptera, Bruchidae). *Zool. Sci.* 16, 955-962.

Molecular Ecology Resources Primer Development C (2011). Permanent Genetic Resources added to Molecular Ecology Resources Database 1 August 2010 – 30 September 2010. *Mol. Ecol. Resour.* 11, 219-222.

O’Neill, S. L., Giordano, R., Colbert, A. M. E., Karr, T. L., and Robertson, H.M. (1992). 16S rRNA phylogenetic analysis of the bacterial endosymbionts associated with cytoplasmic incompatibility in insects. *Proc. Natl. Acad. Sci. U.S.A.* 89, 2699-2702.

Tseng, S. P., Darras, H., Lee, C. Y., Yoshimura, T., Keller, L., and Yang, C. C. S. (2019). Isolation and characterization of novel microsatellite markers for a globally distributed invasive ant *Paratrechina longicornis* (Hymenoptera: Formicidae). *Eur. J. Entomol.* 116, 253-257. DOI: 10.14411/eje.2019.029

Zhou, W., Rousset, F., and O'Neill, S. (1998). Phylogeny and PCR-based classification of *Wolbachia* strains using wsp gene sequences. *Proc. R. Soc. Lond., B, Biol. Sci.* 265, 509–515.
